# Dynamics of inter-farm transmission of highly pathogenic avian influenza H5N6 integrating vehicle movements and phylogenetic information

**DOI:** 10.1038/s41598-021-03284-x

**Published:** 2021-12-17

**Authors:** Dae-Sung Yoo, Byung chul Chun, Younjung Kim, Kwang-Nyeong Lee, Oun-Kyoung Moon

**Affiliations:** 1 Department of Public Health, College of Medicine, Korea University, Seoul, Republic of Korea; 2 Veterinary Epidemiology Division, Animal and Plant Quarantine Agency, Gimcheon, Republic of Korea; 3 Department of Preventive Medicine, College of Medicine, Korea University, Seoul, Republic of Korea; 4 Department of Infectious Diseases and Public Health, Jockey Club College of Veterinary Medicine and Life Sciences, City University of Hong Kong, Kowloon, Hong Kong China; 5 Avian Influenza Research and Diagnostic Division, Animal and Plant Quarantine Agency, Gimcheon, Republic of Korea; 6 Import Risk Assessment Division, Animal and Plant Quarantine Agency, Gimcheon, Republic of Korea

**Keywords:** Epidemiology, Phylogenetics

## Abstract

Highly pathogenic avian influenza (HPAI) in poultry holdings commonly spreads through animal trade, and poultry production and health-associated vehicle (PPHaV) movement. To effectively control the spread of disease, it is essential that the contact structure via those movements among farms is thoroughly explored. However, few attempts have been made to scrutinize PPHaV movement compared to poultry trade. Therefore, our study aimed to elucidate the role of PPHaV movement on HPAI transmission. We performed network analysis using PPHaV movement data based on a global positioning system, with phylogenetic information of the isolates during the 2016–2017 HPAI H5N6 epidemic in the Republic of Korea. Moreover, the contribution of PPHaV movement to the spread of HPAI was estimated by Bayesian modeling. The network analysis revealed that there was the relationship between phylogenetic clusters and the contact network via PPHaV movement. Furthermore, the similarity of farm poultry species and the shared integrators between inter-linked infected premises (IPs) were associated with ties within the same phylogenetic clusters. Additionally, PPHaV movement among phylogenetically clustered IPs was estimated to contribute to approximately 30% of HPAI H5N6 infections in IPs on average. This study provides insight into how HPAI spread via PPHaV movement and scientific basis for control strategies.

## Introduction

Over the last decade, the rapid spread of animal infectious diseases has often been observed, mainly driven by highly interconnected livestock productions through industrialized supply chains. This is true for highly pathogenic avian influenza (HPAI) disease at poultry holdings. HPAI is a viral notifiable infectious disease (per World Organisation for Animal Health) with a high mortality rate in chickens. It commonly spreads through contact between an infectious and susceptible hosts. Indeed, an increasing number of HPAI virus (HPAIV) infections have been reported at poultry facilities globally, in part due to increased intensification and complexity of interactions despite enormous efforts to eliminate and prevent the disease worldwide^1^.

To effectively prevent and control the spread of HPAIV, understanding the contact structure between different poultry holdings at risk is essential. For example, poutlry transport between farms are potential spreads of HPAIV. Additionally, indirect contact with fomites through vehicle movement associated with production and health, such as feed trucks and egg transporters visiting IPs, could spread infections between farms^2^.

Recently, many studies have identified the role of animal movement in transmitting a pathogen between livestock farms using network analysis. Previous network studies have identified a relationship between contact properties of poultry farms and HPAI occurrence by comparing network metrics; for example infected premises were more likely to have a higher degree of centrality and betweenness centrality^3,4^. More recently, one study introduced a permutation approach to evaluate the intermediary role of a particular link or node in the contact network on the spread of pathogen on a community level^5^. These studies have provided important knowledge on the effect of contact structure between farms on HPAI spread.

However, despite the importance of understanding transmission chains via vehicle movement, epidemiological studies on the impact of vehicle movement IPs and susceptible premises remains scarce, mainly owing to a lack of corresponding data. With the aim of tracing epidemiological links to allocate biosecurity and surveillance resources^6^, the Republic of Korea (ROK) has started mandatory registration regulations for all vehicles visiting livestock productions for production and health-associated duties since 2014, which are attached to a global positioning system (GPS) to prevent and control infectious disease. Up to date, 18 kinds of livestock vehicles such as feed trucks, livestock transporter trucks, livestock manure hauling trucks, and egg delivery trucks transmit every farm visiting record to the Korea Animal Health Integrated System (KAHIS). With the real-time movement tracking data, it is possible to estimate the model for the spread of HPAIV between farms due to vehicle movement. This will contribute to our understanding of HPAI transmission among poultry farms and therefore improve the efficiency of intervention resource allocation to poultry productions.

Additionally, as the HPAIV undergoes an evolutionary genetic change during the course of an outbreak, phylogenetically different genotypes of the virus can be isolated from infected hosts (about 91.5–94.1%, for homologies of the PA gene, 96.7–97.3% for homologies of the NS genes among the five genotype groups)^7^. Therefore, phylogenetic clustering information on IPs can be used to infer more accurate transmission dynamics between poultry holdings^8^.

Hence, this study aimed to elucidate the transmission dynamics of HPAI H5N6 via poultry production and health-associated vehicle (PPHaV) movement by integrating GPS tracking data and phylogenetic information during the 2016–2017 HPAI H5N6 epidemic in the ROK. First, we extracted potentially contaminating vehicle movements based on various temporal assumptions on the infectious duration of IPs and PPHaV movements. Subsequently, an inter-farm contact network was constructed based on those movements and phylogenetic information. We conducted a network analysis to elicit the association between the HPAI H5N6 transmission chain and PPHaV movements. Second, we quantified the contribution of PPHaV movements on HPAI H5N6 occurrence in IPs and the length of time for infectious duration followed by a permutation test to evaluate distinctive characteristics of contact networks among IPs with the same genotype virus infection. This study provides novel insight on the dynamics of inter-farm spread via a diverse range of vehicle movements and a scientific basis for HPAI control strategies including movement restriction and standstill.

## Results

### Network with all genotypes of IPs

To examine the rate at which HPAI H5N6 transmission between poultry farms is driven PPHaV movement, we used a network metric named assortativity. This denotes how likely IPs with the same genotype of HPAI H5N6 are to be connected to each other. We measured this indicator on the contact network built with tie formations via PPHaV movement using the different temporal infectious duration assumption for vehicle and IPs.

Table 1 summarizes the characteristics of networks composed of all genotypes of IPs grounded on the different temporal assumptions of the infectious duration for IPs (i.e., seven days, 14 days, 21 days) and PPHaV movements (i.e., one day and three days). The counts of nodes comprising the network tended to increase as the infectious period for PPHaV increased from one day to three days and as the infectious durations for IPs increased. For example, the number of IPs as nodes increased from 80 during the seven-day infectious duration of IPs to 130 during the twenty-one-day infectious duration of IPs with a one-day infectious period for PPHaV movement; this increased further to 195 during the twenty-one-day infectious duration of IPs with a three-day infectious period for PPHaV movement. The farm species and genotype composition of IPs in each network were relatively comparable in all scenarios.Chicken, duck , and quail farms accounted for 57.4%, 41.1%, and 1.4% of IPs as nodesThe different genotypes C2, C3, C4, and C5 accounted for 26.8%, 19.1%, 46.0%, and 8.1% of IPs.

**Table 1:** Overview of the characteristics of the contact networks between all HPAI H5N6 genotypes of infected premises.

Infectious duration for vehicle	One day			Three days		
Infectious duration for IPs	7 days	14 days	21 days	7 days	14 days	21 days
**Network element**						
No. of unique nodes (IPs)	80	113	129	130	176	195
No. of edges between IPs	100	223	369	251	603	978
No. of unique edges between IPs	78	158	233	147	370	498
**Genotype of IPs**						
No. of C2	19 (23.8%)	30 (26.5%)	33 (25.6%)	34 (26.2%)	51 (29.0%)	58 (29.7%)
No. of C3	17 (21.3%)	22 (19.5%)	26 (20.2%)	24 (18.5%)	31 (17.6%)	34 (17.4%)
No. of C4	38 (47.5%)	52 (46.0%)	59 (45.7%)	61 (46.9%)	80 (45.5%)	87 (44.6%)
No. of C5	6 (7.5%)	9 (8.0%)	11 (8.5)	11 (8.5%)	14 (8.0%)	16 (8.2%)
**Farm species of IPs**						
No. of chicken farms	45 (56.3%)	70 (61.9%)	78 (60.5%)	70 (53.8%)	98 (55.7%)	110 (56.4%)
No. of duck farms	34 (42.5%)	42 (37.2%)	48 (37.2%)	58 (44.6%)	76 (43.2%)	82 (42.1%)
No. of quail farms	1 (1.3%)	1 (0.9%)	3 (2.3%)	2 (1.5%)	2 (1.1%)	3 (1.5%)
**Network metrics**						
Density	0.011	0.011	0.012	0.009	0.011	0.013
Assortativity by genotype	0.743	0.666	0.665	0.616	0.538	0.547

The networks were built based on the three different infectious duration for infected farms and two infectious durations for potentially contaminating vehicle movements.

*HPAI* highly pathogenic avian influenza, *IP* infected premises.

**Figure 1:**
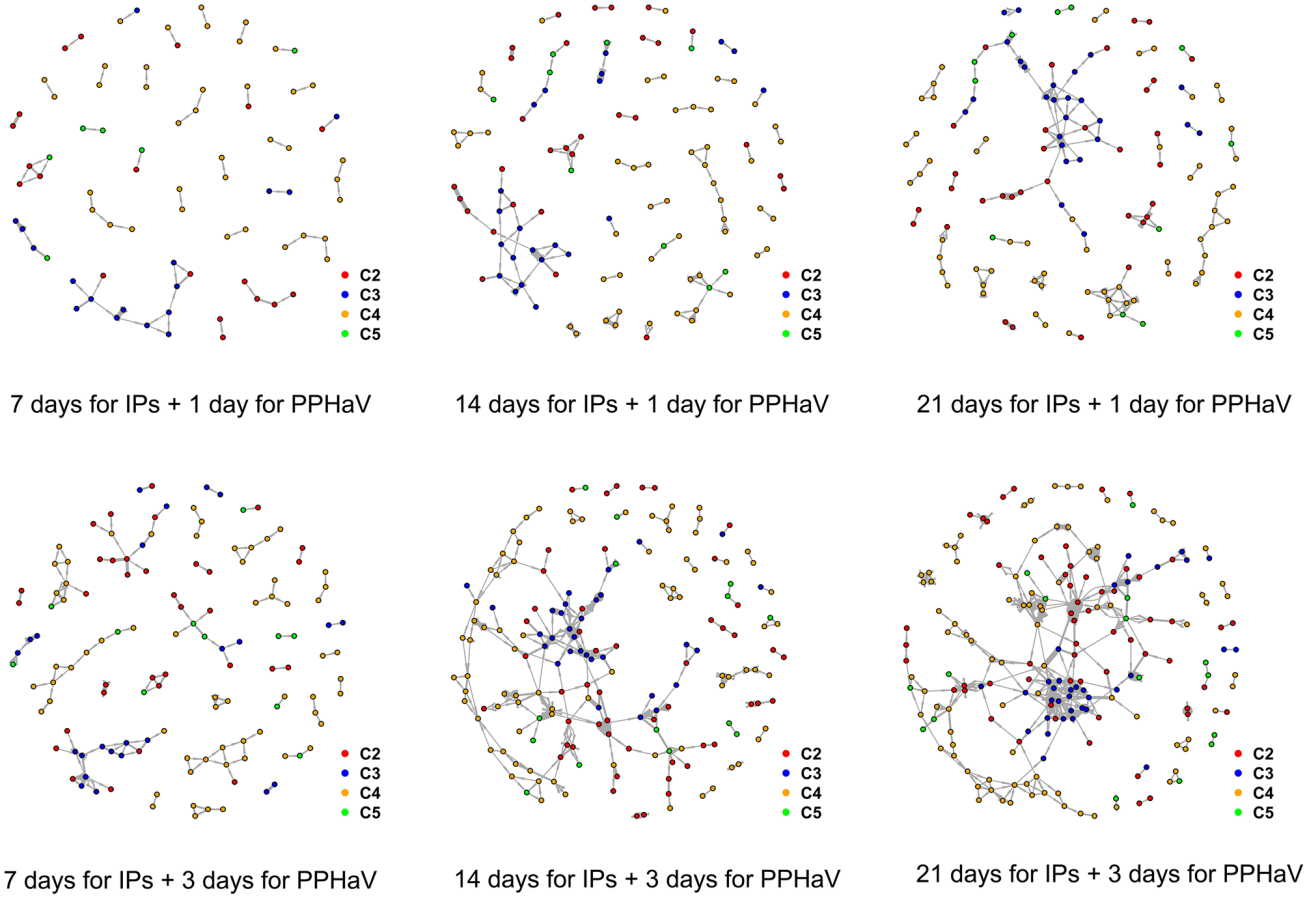
Graphical representation of networks consisting of all infected premises (IPs), constructed by under different temporal assumption on infectious duration for IPs and poultry production and health associated vehicle (PPHaV) movements**.** Each of figures represent the contact network between IPs, under the different infectious duration for IPs and vehicle movement. The plots in the top row display one day infectious duration for vehicle movement with seven days infectious duration for IPs (top left), 14 days duration (top middle), and 21 days duration for IPs (top right). The plots in the bottom rows depict three days duration for vehicle movement with seven days infectious duration for IPs (bottom left), 14 days duration (bottom middle), and 21 days duration for IPs (bottom right). Colored dot denotes specific genotype IPs; red for C2, blue for C3, yellow for C4 and green for C5.

For the assortativity of six contact networks based on the genotype of IPs, all had more than about 0.53, which indicated the tendency to connect IPs with other IPs having the same genotype HPAIV via potential contaminating vehicle movement. The assortativity was generally higher in the network built with an infectious duration of one day for PPHaV movements than in the network that used the three-day assumption. More specifically, the contact networks built simulated using a seven-day infectious duration for IPs and a one-day duration for PPHaV movement showed the highest assortativity by genotype. This implies that the contact structure made by a more temporary potential contaminating vehicle movement between IPs having a shorter infectious period was more cohesive and likely to be the possible transmission chain of the HPAIV H5N6. Indeed, the majority of the same genotype of IPs were directly or closely interconnected with each other, while a few were connected with other genotypes of IPs, as shown in Fig. 1.

Additionally, we performed logistic regression quadratic assignment permutation (LR-QAP) analysis to identify factors contributing to tie formation between the interlinked two IPs with the same genotype of HPAI H5N6 infection via PPHaV movement. Table 2 provides the results of the LR-QAP analysis of the association between tie formation among the same genetic group of IPs and dyadic explanatory variables (i.e., pairwise variables between two interlinked infected premises) with different temporal assumptions for the contagious period for IPs and PPHaVs. Overall, coefficient estimates exhibited a significant positive association among the similarity of farm species (minimum[min]-maximum[max], 6.57 to 9.25), shared poultry integrators (min–max, 0.98 to 2.15), and tie presence. In contrast, the geographical distance of dyadic nodes, Euclidean distance between interlinked infected premises measured as a continuous scale, was significantly inverse associated with it (min–max, − 0.06 to − 0.01) except for one network with a seven-day infectious duration for IPs and PPHaVs. In other words, given that both poultry integrator and farm species between interlinked infected premises via vehicle movements are the same, the estimated probability of a tie formation between HPAI H5N6 virus-infected premises for a 1 km increase in geographical distance between two farms reduced. LQ-QAP analysis showed the lowest Bayesian information criterion (BIC) value for seven-day infectious periods for IPs and one-day periods for PPHaVs.

**Table 2:** Association between three dyadic explanatory variables and a tie formation between the same genotype IPs under the different infectious duration for infected premises and vehicle movements using logistic regression quadratic assignment procedure (LQ-QAR).

Infectious duration for IPs + PPHaV	Coefficient estimates (*p* value)			BIC
	Farm Species (ref. = different)	Poultry integrator (ref. = different)	Spatial distance (unit = km)	
7 days + 1 day	9.25 (0.002)	1.69 (0.031)	− 0.03 (0.274)	103.152
14 days + 1 day	8.46 (0.004)	1.60 (0.009)	− 0.06 (< 0.001)	181.382
21 days + 1 day	7.48 (0.003)	2.15 (0.012)	− 0.03 (0.001)	305.840
7 days + 3 day	7.81 (0.002)	0.98 (0.038)	− 0.01 (0.040)	309.821
14 days + 3 day	6.96 (0.004)	1.31 (0.010)	− 0.03 (0.022)	585.492
21 days + 3 day	6.57 (0.040)	0.99 (< 0.001)	− 0.03 (< 0.001)	889.949

*IPs* infected premises, *PPHaV* poultry production and health associated vehicles, *ref* reference, *BIC* Bayesian information criterion.

### Bayesian inference

To estimate the contribution of vehicle movements originating from 259 HPAI H5N6 virus IPs of 343 IPs to spreading HPAI H5N6 virus, we conducted Bayesian inference to estimate the parameters associated with the force of infection of vehicle movements and other factors. Table 3 summarizes the estimated contribution rate of introducing a specific genetic type of HPAIV H5N6 to an individual farm via PPHaV movements originating from another IP or other sources of infections through the simulation using the force of infection parameters previously estimated by Bayesian inference. The posterior parameters for force of HPAI H5N6 infection due to one contaminating PPHaV entry coming from IPs was estimated to differ with the infectious periods of the genotype of IPs and vehicle movement (see Supplementary Table S3 and Fig S2–S3 online). The deviance information criterion (DIC) was decreased in the model employing a three-day infectious duration for vehicle movements except in the case of C3-genotype HPAIV H5N6 infection, where the one-day infectious duration model was preferred (see Supplementary Table S3 online).

**Table 3:** Contribution rate of potentially contaminating vehicle movement and other sources of HPAI H5N6 infection at infected premises that belong to each phylogenetic cluster.

Parameter	HPAI H5N6 genotype cluster			
	C2 (no. of IPs = 81)	C3 (no. of IPs = 41)	C4 (no. of IPs = 114)	C5 (no. of IPs = 23)
**Proportion of transmission route (%)**				
Vehicle movement	23.5%	31.7%	46.0%	11.8%
Other sources	76.5%	68.3%	54.0%	88.2%
Interval between infection and reporting (days)	5.89 (1.01, 12.87)	5.90 (1.00, 12.88)	5.98 (1.00, 12.94)	5.87 (1.00, 12.85)
**Duration of vehicle infectious (days)§**	3	1	3	3
No. of vehicles	317	125	326	123
No. of vehicle movements	2,173	523	2,958	913
Between IPs	230	130	529	13
IPs to non-IPs	1,943	393	2,429	900

With the force of infection was calculated based on the posterior marginal distributions of each parameter, the proportion of potential route of transmission contributing to HPAI H5N6 infection in IPs that belong to a specific phylogenetic cluster was obtained by simulating a binomial trial with two possible outcomes (e.g., vehicle movement and other sources of infection) to compute.

The distribution was obtained by simulating values from the Gamma distribution, based on parameters α and β randomly sampled from their joint distribution.

Based on DIC value, the preferred model between one day and three-day infectious duration for vehicle movements originating from a specific genotype of IPs was selected.

*HPAI* highly pathogenic avian influenza, *IPs* infected premises, *DIC* deviance information criterion.

Overall, 259 IPs (75.5% of all IPs) with both a visit log for GPS-equipped vehicles and phylogenetic classification information and 6,280 non-IPs were used to estimate the contribution of potentially contaminating movements to HPAI H5N6 transmission between poultry premises. Apparently, 38.7% of IPs that had visiting records from registered vehicles were directly interlinked with other IPs. The proportion of HPAI H5N6 spread via potentially contaminating vehicle movement differed with the genotype of IPs related to potentially contaminated vehicle movement in preferred models. On average, 28.3% of 259 IPs were transmitted HPAIV H5N6 via PPHaV movement in which 13.7% of interconnection occurred between IPs and 86.3% of links occurred between IPs and non-IPs, whereas 71.7% of 259 IPs were given HPAIV H5N6 by other sources. When C4-genotype IPs were associated with potentially contaminated vehicle movement, 46.0% of IPs had an infection with three-day infectious PPHaV entries; in contrast, 31.7% of poultry holdings had an infection via C3-genotype IPs associated with potentially contaminated vehicle movement that were presumed to transfer virus for one days. Moreover, the IPs origin vehicle movement that stayed infectious for three days were attributed to HPAI H5N6 infection for 23.5% of C2-genotype of IPs and 11.8% of C5-genotype of IPs. In addition, the interval between infection and reporting or sampling was estimated to be 5.91 days (5.89 days in the C2 IPs, 5.98 days in the C3 IPs, and 5.87 days in the C4 IPs).

**Figure 2:**
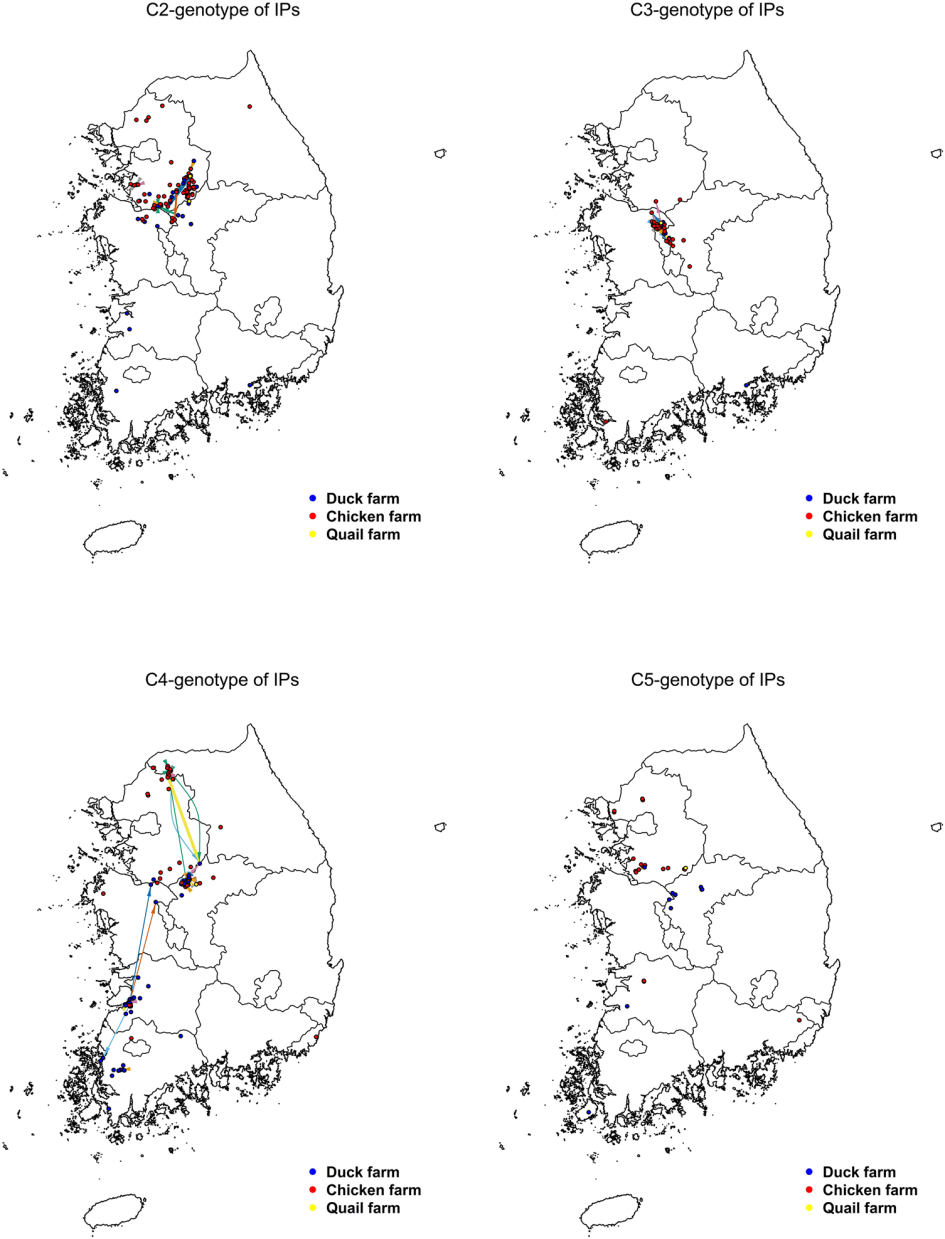
The geographical contact network between the same HPAI H5N6 genotype of infected premises (IPs) constructed under temporal infectious duration of IPs and vehicle movement based on Bayesian inference; C2(top left), C3(top right), C4(bottom left) and C5(bottom right). Blue dot denotes a duck farm, red dot points out a chicken farm and brown dot represents quail farm. The color of edges represented an individual vehicle movement.

Furthermore, as shown in Fig. 2, there were spatially heterogeneous contact patterns between the same genotype of IPs via vehicle movements. A majority of contacts were established in the central part of the country via PPHaV movements between infected poultry holdings at a close distance. The long-range contacts were only present in the C4 genotype of IPs, located in the northern, central, and southern parts of the country, were interconnected via PPHaV movements.

### Genotype-specific network based on Bayesian inference

To explore contact properties relative to HPAI transmission via IPs origin vehicle movements, we examined six contact properties on the simulated contact network composed of only the same genotype IPs based on the parameter estimates from Bayesian inference: diameter (the shortest distance between the two most distant nodes in the network), average path length (the number of ties along the shortest length of the tie between two nodes), density (the proportion of edges that exist in a network), reciprocity (the proportion of IPs in a directed network to be mutually linked), assortativity, and transitivity (the ratio between the observed closed triplets and the maximum possible number of closed triplets in the network). Table 4 describes the results of the permutation analysis on the contact network between the same genotype of IPs constructed by potentially contaminating vehicle movement originating from IPs with infectious duration based on the Bayesian inference results. Each of the three networks had different compositions of chicken farms and domestic duck farms. For instance, C2 and C3 genotype-based networks had a high proportion of chicken farms: 100% of IPs raised chickens in the C3 genotype-based network, and 78.9% of IPs were domestic chicken farms in the C2 genotype-based network whilst the chicken holdings in the C4 genotype-based network accounted for 26.9% of IPs.

**Table 4:** Permutation test of contact network structure metrics between the same genotype of HPAI H5N6 virus infected premises linked via vehicle movements based on the Bayesian parameter estimation.

Network metrics	C2 (No. of IPs = 19 (chicken 15, duck 4), Edge density = 0.051)		C3 (No. of IPs = 13 (chicken 13), Edge density = 0.109)		C4 (No. of IPs = 52 (chicken 14, duck 38) Edge density 0.020)	
	Observed value	Median difference (5–95 PCTL)	Observed value	Median difference (5–95 PCTL)	Observed value	Median difference (5–95 PCTL)
Assortativity by species	1.000	–	NA §	–	0.820	–
Diameter	5	1 (− 3, 3)	5	− 1 (− 4,2)	4	− 4 (− 11, 0)
Average path length	1.071	− 0.771 (− 2.191, − 0.116)	1.653	− 1.007 (− 1.871, − 0.063)	1.258	− 1.825 (− 4.192, − 0.542)
Reciprocity	0.286	0.286 (0.055, 0.286)	0.348	0.257 (0.062, 0.348)	0.231	0.231 (0.151, 0.231)
Assortativity by degree	0.417	0.473 (− 0.003, 0.949)	0.634	0.693 (0.315, 1.094)	0.068	0.084 (− 0.207, 0.347)
Transitivity	0.600	0.600 (0.284, 0.600)	0.200	0.006 (− 0.146, 0.2)	0.333	0.301 (0.229, 0.333)

Networks for permutation test were generated based on the observed number of IPs (nodes) and probability of edge formation between nodes, which was calculated by the observed edge density.

MD, median difference between observed value and 1000 simulated values; 5–95 PCTL 5–95% percentile range of difference.

NA, not available; because all IPs were chicken holdings, the corresponding assortativity were not available.

Diameter is the shortest distance between the two most distant nodes in the network. Average path length is equal to the number of ties along the shortest length of the tie between two nodes. Reciprocity denotes the proportion of nodes in a directed network to be mutually linked. Transitivity refers to the ratio between the observed closed triplets and the maximum possible number of closed triplets in the network.

HPAI, highly pathogenic avian influenza; IPs, infected premises.

The three networks were commonly characterized by a significantly short average path length and high reciprocity. In contrast, the reciprocity, assortativity by degree, and transitivity showed different patterns in the three networks (see Supplementary Figs S8–S10 online). More specifically, the C3 genotype-based network, which was solely composed of chicken farms, had a significantly high assortativity by degree (median difference between the observed value and 1,000 simulated values [MD], 0.634; 5–95% percentile range of difference [5–95 PCTL], 0.315–1.094). In contrast, the contact network between the C2 genotype of IPs and C4 genotype of IPs, consisting of domestic duck and chicken farms, exhibited significant high transitivity; the median of transitivity and reciprocity were 0.600 (5–95 PCTL, 0.284–0.600) in the network with C2 genotype IPs and 0.301 (5–95 PCTL, 0.229–0.333) in the network with C4 genotype IPs.

## Discussion

HPAI virus generally spreads through effective contact between a contagious host and a susceptible one. The primary type of contact between poultry holdings is via animal trade and vehicle movement engaging in poultry production and health tasks. Recently, many studies have identified the role of animal movement in transmitting a pathogen between livestock farms. However, very less attention has been paid to examine the association of vehicle movement, such as that of feed trucks and egg transporters. Furthermore, none of the studies have incorporated phylogenetic information for the more reliable estimation of the contributions of vehicle movement between infected premises on viral diffusion. Therefore, this study aimed to provides better understanding of the transmission dynamics of HPAIV H5N6 via vehicle movement and a scientific basis for animal health authorities to refine countermeasures including customized standstills and movement control. In this study, we used PPHaV movement data and phylogenetic classification information to elucidate the role of PPHaV movement on inter-farm transmission of HPAI H5N6 and to understand the structural properties of contact networks among IPs.

In the network analysis on contact structure via PPHaV movement among all HPAI H5N6 genotypes of IPs, we first identified a higher preference of bonds between the same phylogenetic group of IPs rather than a connection between different genotypes of IPs. This finding suggests that there is a correlation between the interconnection of the same genotype of IPs and PPHaV movement that potentially spreads the virus through the network. This emphasizes the importance of epidemiological tracing utilizing vehicle tracking data that elucidate effective surveillance and preventive actions.

Moreover, when tracing epidemiological linkages to search for potentially high-risk IPs and farms, animal health authorities should consider the various lengths of time for tracing to harness the efficiency of biosecurity resource allocation. Under current standard operating procedures (SOP) for responding to HPAI outbreaks in the ROK, all poultry farms that PPHaVs have visited after entering an IP during the 21 days prior to the reporting date are obliged to take tests and undergo poultry movement restriction. Although the authorities are responsible for minimizing the possibility of poultry farms being infected by vehicle movement from IPs to eliminate epidemics rapidly, prioritizing biosecurity and surveillance targets is important, especially in the acceleration phase of the epidemic.

In permutation analysis of all the IP networks, similarities of farm poultry species, shared poultry integrators, and closer geographical distance between dyadic interlinked IPs were identified as contributing factors to building potential transmission routes. Over the last decade, poultry integrators have been profoundly rooted in the poultry production system in the ROK. According to the monthly reports from the Korean Poultry Association^9^, approximately 90% of meat duck farms and 91% of meat chicken farms contracted with integrators; these farms share production chains such as feed suppliers, chick sources, and duck slaughtering plants. In addition, poultry production-associated vehicles, such as feed trucks and manure haulers, are generally responsible for farms in certain areas. Therefore, the poultry holdings in proximity are more likely to be interconnected by those shared vehicles (see Supplementary Fig S11 online). The consistency of farm poultry species between IPs seems to be a fundamental driver of tie formations because most PPHaVs can be separately operated by the type of poultry species at the farms they visit mainly due to structural differences in car design and supply lines of resources^4^. This implies that targeted standstill of poultry holdings located at an adjacent distance that raise the same species and are operated by the same poultry integrator of IPs could effectively reduce further spread through the networks.

Using Bayesian inference, the HPAI H5N6 virus were likely to introduce 28.3% (minimum–maximum, 11.8%–46.0%) of IPs via possible infectious vehicle movements originating from the same phylogenetically clustered IPs. This could be explained by the finding in the permutation test on network structure metrics. The three contact networks between the same genotype IPs depicted significantly high cohesive connection structure, such as high transitivity, reciprocity and low average path length which is suitable for disease transmission via vehicle movements. This means if one poultry holding have infection and continue to production without early detection, rest of other premises are easily affected by through the network around an IP. Furthermore, the contact structure is potentially correlated with the composition ratio of poultry holdings and the geographical distance between them. For instance, the C3 genotype-based networks consisting only of chicken farms exhibited increased reciprocity pertaining to PPHaVs that had repetitively visited two paired chicken farms showed higher reciprocity than species heterogenous network. This indicated that the contacts between IPs tended to be direct farm-to-farm contacts rather than overlaid interactions between farms via vehicle movement. In contrast, the C2 genotype-based contact network that included chicken and duck farms located in neighboring regions had high transitivity and reciprocity. High transitivity means that if one poultry holding has an HPAIV H5N6 infection and continues to produce without detection, other interlinked premises are easily affected by an IP. In this type of relationship, inter-farm transmission of HPAI H5N6 could be prevented by enhancing on-farm biosecurity practices for PPHaVs and exterior personnel.

As states in the epidemiological report for the HPAI epidemic in ROK^10^, HPAI viruses were isolates from samples of shoes, vehicles, and entry ground in the infected premises. This indicated that the shoes of the driver of vehicles were likely to be contaminated. This suggests the transmission between infected premises through indirect contact with farmworkers and poultry. This is supported by a previous study showing that the disinfection of personnel visiting farms reduced the risk of Avian influenza infection in poultry farms^11^. In this context, drivers and poultry producers moving around poultry productions should implement stricter disinfection protocols, including disinfection after cleaning removes dirt, dust with longer contact time on both the vehicle itself and the equipment such as shoes and clothes.

**Figure 3:**
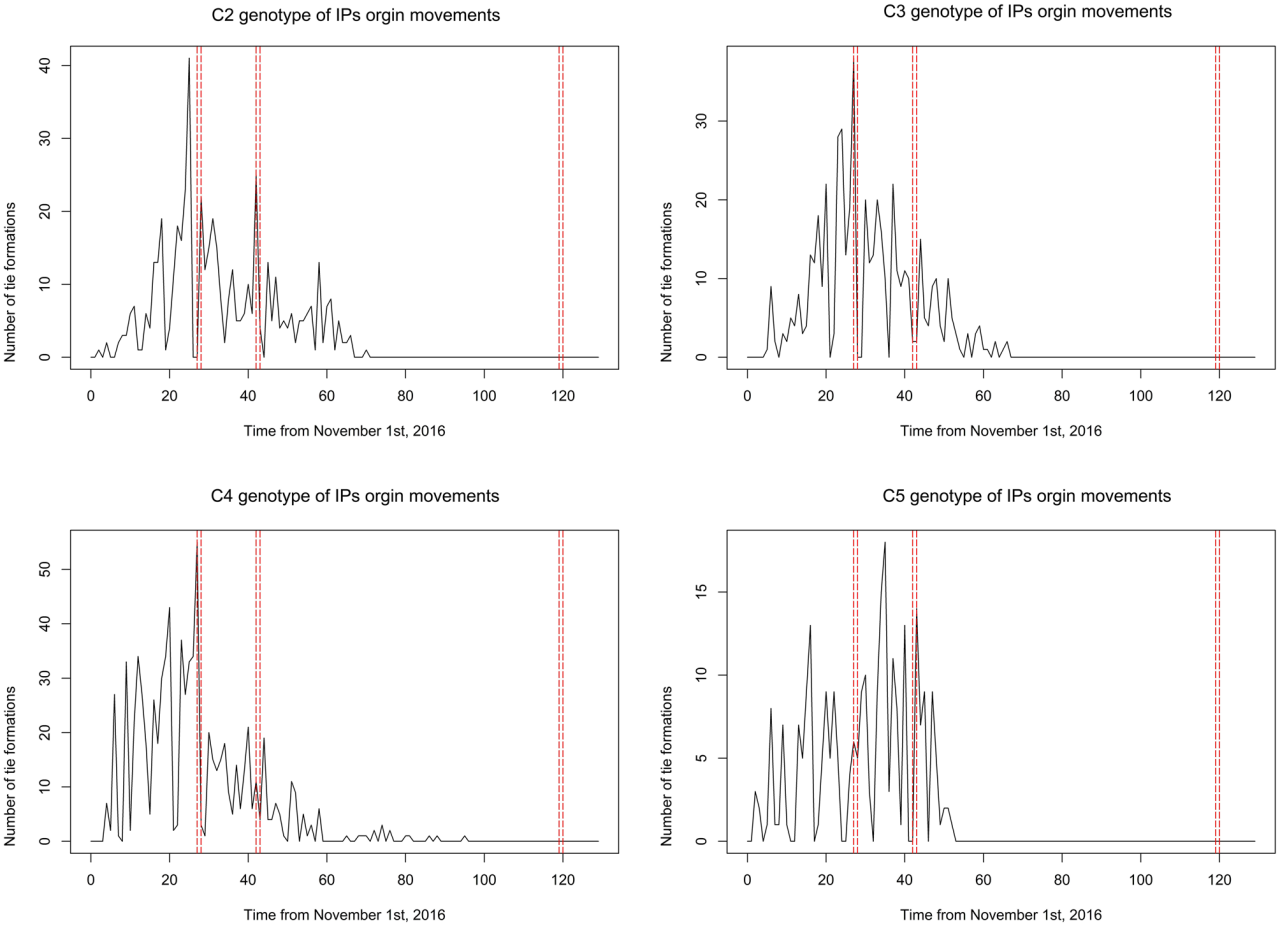
The temporal trends of tie formations with HPAI H5N6 infected premises (IPs) via potentially contaminating vehicle movement that was presumed to infectious duration of one day; C2(top left), C3(top right), C4(bottom left) and C5(bottom right). Red dashed vertical lines represent the period of standstills; First standstill was enforced on ten municipalities starting from November 28th, 2016 to November 29th, 2016. Second one was nationwide from December 13rd, 2016 to December 14th, 2016 and third one was applied to three municipalities from February 28th, 2016 to March 1st, 2017 (time unit: day).

Besides, the common types of vehicles observed in the three networks were feed trucks and egg transporters (Table 5). Interestingly, the proportion of egg transporters (7.1–17.1%) that could be involved in potentially infectious routes was pronounced compared to its proportion of the total number of registered PPHaV (4.4%) as of March 2019. Egg transporters enter inside layer farms to load many eggs almost on a daily basis. During the process of loading eggs, egg transporters are prone to direct contact with farm workers, especially in small-scale businesses. Furthermore, the risk of HPAI H5N6 infection can increased by transporting egg trays used to remove eggs during an epizootic^12^. Therefore, egg delivery vehicles and personnel are more likely to be contaminated with viruses when the premise is infected. In addition, the length of the interval between the infected and reporting dates is parallel to the duration when the highest assortativity value occurred. The temporal interval between the infected and reporting or sampling dates was estimated to be 5.91 days on average. In practice, disease detection speed on poultry holdings seems to be a condition that depends on the intensity of nationwide surveillance and participation in a voluntary notification. During the 2016–17 HPAI H5N6 epidemic, mandatory testing was proactively implemented in the ROK. As a result, approximately 45% of all infected duck holdings were screened by surveillance. Furthermore, three standstills at the national level or on a broader scale were imposed by an animal health authority. As shown in Fig. 3, the number of tie formations via IP origin PPHaV movements dramatically decreased after a standstill that ordered all PPHaV drivers to initiate disinfection protocols. Thus, a control strategy could result in decreased infectious duration of the vehicle contaminated by fomites.

**Table 5:** Summary of the type of vehicle interconnecting between the same HPAI H5N6 virus genotype of infected premises based on Bayesian parameter inference results.

Genotype of IPs	C2	C3	C4	C5
Infectious duration of IPs + PPHaVs	6 days + 3 days	6 days + 1 day	6 days + 3 days	6 days + 3 days
**Type of vehicle interconnecting IPs (%)**				
No. of feed transporter	12 (85.7%)	13 (56.5%)	31 (59.6%)	
No. of animal transporter		1 (4.3%)	7 (13.5%)	
No. of manure hauling		4 (17.4%)		
No. of egg transporter	1 (7.1%)	4 (17.4%)	5 (9.6%)	
No. of private Veterinarian				1 (100%)
No. of veterinary medicine vendor			3 (5.8%)	
No. of others	1 (7.1%)	1 (4.3%)	6 (11.5%)	

*HPAI* highly pathogenic avian influenza, *IP* infected premises, *PPHaV* poultry production and health associated vehicle.

This study has several limitations. First, we did not account for the risk variance based on the type of vehicles interconnecting between two IPs because of the absence of exact specifications about the purpose of their visits to farms; this was not enough information to estimate parameters relative to the force of infection. In principle, the risk of contamination with HPAI H5N6 differs by the type of vehicle. For instance, bulk feed trucks regularly undergo disinfection procedures after unloading and loading the feedstuff at the feed factory and are unlikely to have direct contact with poultry, while poultry transporters are likely to have direct contact with poultry. Moreover, although we reported the counts of different types of vehicle movements engaged in potential transmission routes to intuitively evaluate the size of the impact of each vehicle, gauging the variance of the contribution of individual types of vehicles to HPAI H5N6 infection at poultry farms is necessary. Furthermore, the variability of other factors including the duration of contamination and underlying risk associated with contact with poultry should be evaluated in a future study. Second, the vehicle movements in our analysis did not encompass all types of vehicles, such as personal owner vehicles, moving around poultry holdings because of privacy issues. Furthermore, there could be missing data of movements, such as broken GPS devices or other reasons. Third, we did not consider the on-farm biosecurity level, which might affect the contribution of vehicle movement to HPAI H5N6 transmission. The risk of a vehicle transmitting the virus likely depends on whether a disinfection procedure was performed. For example, the force of infection is variable between the following two situations: when a vehicle visits a farm after visiting an IP and when a vehicle visits a farm after other farms. Therefore, the extra parameters to reflect the on-farm biosecurity level would be incorporated to more robust estimation of force of infection given by IP origin vehicle entries. Lastly, even though the phylogenetic cluster information used in the study can distinguish and separate the inter-farm transmission linkage among the same genotype of IPs, which was the essential assumption on the network analysis and Bayesian inference, it had the limitation of ruling out completely indirect transmission between IPs. For instance, if one IP had a direct connection with another IP of the same phylogenetic clustering group via vehicle movements, which turned out to connect with different IP genetically directly, the force of infection estimated in the first place would be misleading thus should be excluded. Thus, an alternative approach to incorporating genetic data, including genetic distance from the whole genome sequence of isolates, is needed to attempt future works.

During the 2016–2017 Korean epidemic, although the length of time from infection to reporting was estimated to be short and several movement stand-still periods were enforced, a large number of vehicles visited IPs during their estimated infection period and could have disperse HPAIV H5N6 to other farms. Moreover, our study demonstrated the presence of long-range viral transfer between IPs via vehicle movements. PPHaVs were required by the regulation to be disinfected at multiple sites during the epidemic in addition to routine cleaning and disinfection procedures at the farm entrance. Our findings suggest that such disinfection procedures may not have been sufficient, possibly due to the tighten interconnection between farms. Therefore, effective restrictions on vehicle movements and risk-based surveillance need to be imposed through evaluation on the underlying contact network between poultry holdings.

This study firstly demonstrated the contribution of poultry production and health associated vehicle movement to spread HPAIV H5N6 among poultry holdings and underscore the understanding of the contact network that could minimize destructive consequence by providing the essential information about those restrictions to be targeted to premises and risk based active surveillance. It is recommended that an animal health authority should analyze the real-time vehicle movement data to assess the poultry holdings at risk and prevent the further spread.

## Methods

### HPAI outbreak data

According to the terrestrial manual 2018 of the World Organisation for Animal Health Organization (OIE)^13^, HPAI H5N6 virus infection at a poultry holding was diagnosed by the real-time reverse transcription polymerase chain reaction (RT-PCR) test under the active surveillance where the test positive was regarded as viral infection. During the 2016–17 HPAI H5N6 epidemic (from November 16, 2016, to March 3, 2017), the veterinary services conducted a proactive surveillance program in which all duck holdings had to be tested for HPAI H5N6 infection, and chicken holdings received visual inspection every week.

HPAI H5N6 outbreak data contained geographical location, flock size, farm species, contracted integrator, date of HPAIV sampling or reporting, and start and end date of culling for 343 HPAI H5N6 virus-IPs. Moreover, phylogenetic cluster information from a previous study was added to the abovementioned information for each IP^14,15^. In fact, during the 2016–17 HPAI H5N6 epidemic, the H5N6 viruses isolated from poultry farms and wild birds were classified into five distinct phylogenetic clusters (cluster 1 to cluster 5) according to previous studies^7,14,15^. The phylogenetic analyses were conducted using the whole genome sequence of isolates sampled from HPAI positive poultry. Maximum likelihood (ML) trees were constructed using nucleotide substitution models (Hasegawa-Kishino-Yano model with a gamma (γ)-distribution for HA and NP; general time-reversible model with a γ-distribution for PB2, PB1, PA, and NA; Kimura 2-parameter model with a γ-distribution for M; Tamura 3-parameter model with a γ-distribution for NS). The phylogenetic clustering of trees was based on the phylogenetic variations in the PA and NS gene segments where isolates showed district phylogenetic variations, harbored four (PA I to IV) and two (NS I and II), resulting in the classification of H5N6 HPAIVs into five genotypes (C1–C5). Of 343 IPs, the HPAI H5N6 virus isolated from 311 IPs was phylogenetically classified into four different clusters (hereafter genotypes), namely, C2, C3, C4, C5 (C1 only confirmed in wild bird species). Hence, we utilized these genotype classification numbers for individual IPs to categorize HPAI H5N6 outbreaks and distinguish transmission chains. Additionally, 7,954 non-IPs data was collected where the geographical coordinates, flock size, farm species, and the start date of preemptive depopulation were included, if applicable, which was collected as of July 28, 2016. Those data were obtained from the Animal and Plant Quarantine Agency (APQA), ROK. Non-IP was defined as commercial poultry farms having HPAI negative test results during national surveillance from November 16, 2016, to March 30, 2017.

### Between-farm vehicle movement

**Figure 4:**
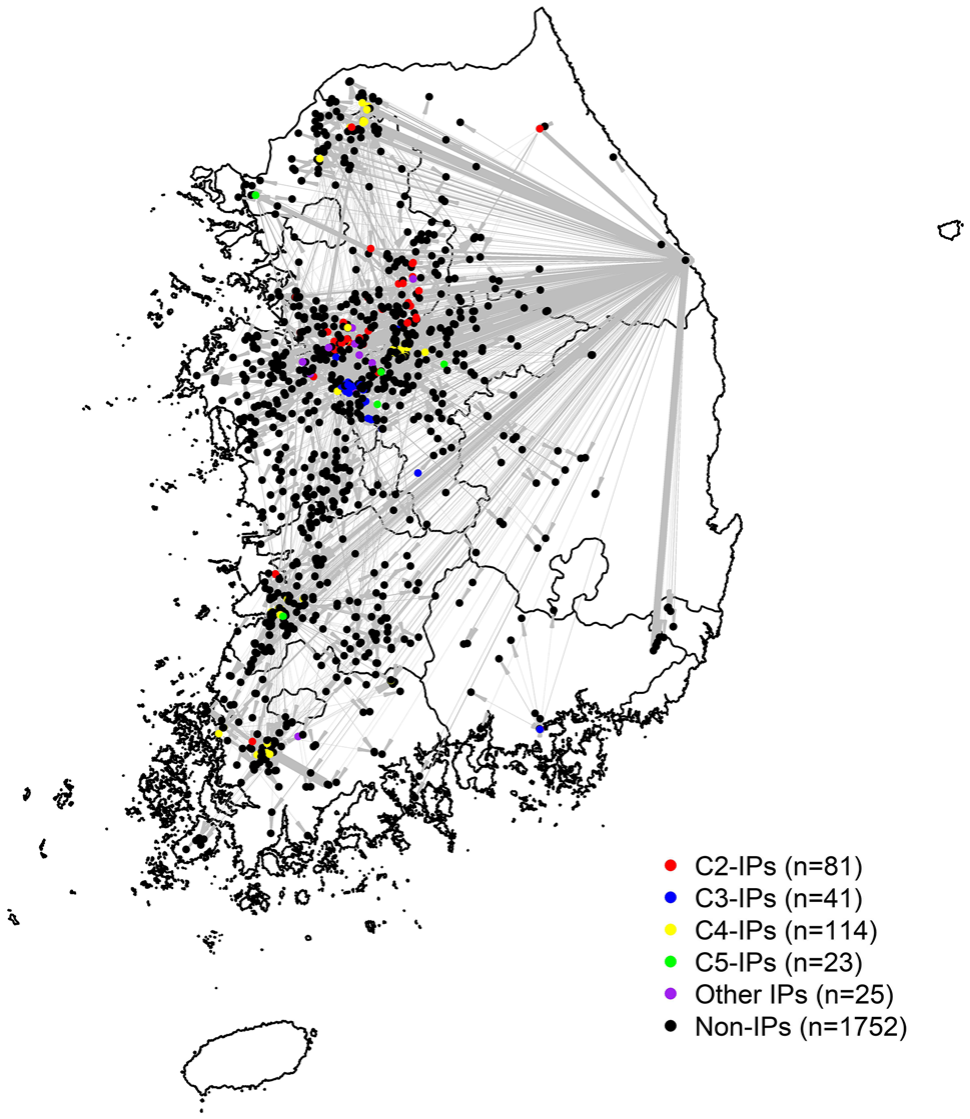
Potentially contaminating vehicle movements originating from HPAI H5N6 infected premises (IPs) during 2016–17 epidemic across the country**.** There were 284 out of 343 IPs origin vehicle movements affecting 2,036 poultry holdings including 1,752 non-infected premises (dots colored with black). Those 284 IPs were classified into four groups based on HPAI H5N6 phylogenetic clusters except for 25 farms (dots colored with purple); 81 IPs belonged to cluster 2 (dots colored with red), 41 belonged to cluster 3 (dots colored with blue), 114 IPs belonged to cluster 4 (dots colored with yellow) and 23 IPs belonged to cluster 5 (dots colored with green).

We obtained PPHaV movement data from the KAHIS, which includes a total 37,424 of visiting records to livestock productions associated with 284 HPAI H5N6 virus IPs of 343 IPs, excluding 59 premises where no visit log of preregistered vehicle movement was recorded as shown in Fig. 4. By law, PPHaVs are required to be installed with a GPS tracking device that transmits a signal to the KAHIS when it is located inside the boundary of a farm registered in the government database. All types of PPHaVs (n = 18) were listed in Supplementary Table S2 online. As of 2019, 59,521 vehicles were registered in the KAHIS (https://home.kahis.go.kr/home/sce/sce_m1_01.do). According to the regular inspection, it can be assumed that the great majority of vehicles are registered. Especially, the registration rate of veterinary service vehicles, feed and manure transporters are considered nearly 100% because the unregistered vehicles are not allowed to enter designated facilities.

PPHaV movement data among poultry holdings were processed as follows. First, all movements originating from 284 IPs by PPHaVs were extracted from KAHIS during the study period from October 27, 2016, (20 days before the first IP report) to March 10, 2017 (one week after the last IP report). We determined study period and the maximum interval between infection and reporting was 21 days as specified in the OIE terrestrial code^13^ following reasons. First the incubation period of HPAI H5N6 infection in domestic poultry is estimated to be approximately four days^16^. Second the domestic duck infected with the HPAI H5N6 virus did not show apparent clinical signs and took a longer time to death while the infected chicken was showing apparently clinical signs^17^. Third, each poultry holding took a weekly mandatory test under nationwide active surveillance with the uncertainty of sensitivity (i.e., false negative) concerning different sample sizes, especially in the low prevalent phase^18^. Additionally, it was supposed that IPs were infectious from one day after the onset of infection and remained infectious until reporting or sampling date, because all both entries and exits from IPs come to be banned immediately after confirmed. Next, we probed inter-farm PPHaV movements from the extracted data that potentially associated with HPAI H5N6 virus transmission. It was presumed that a vehicle contaminated following a visit to an IP in the infectious state was infectious for other poultry holdings during a given period. The vehicle movements meeting this condition was defined as ‘potentially contaminating PPHaV movements’. Although HPAIV is known to be persistent, with the ability to survive for several weeks in the environment^19,20^, given the fact that vehicles are mandated to have disinfection before entering poultry holdings under HPAI control policy, the vehicles contaminated with HPAIV were presumed not to sustain infectivity for longer than three days.

### Network analysis on all IPs

Given potentially contaminating vehicle movements under different assumptions about the length of interval between the infected and reporting days (7 days, 14 days, and 21 days) and the infectious duration for PPHaV movements (one day and three days), we built directed and weighted networks that were only composed of IPs with the genotype confirmed (259 of 284 IPs with visit logs, see Supplementary Fig S12 online ), excluding 25 IPs without phylogenetic cluster information as given in Fig. 4. In the network, each IP was considered a node, and a PPHaV movement between two IPs was regarded as an edge. The network was weighted by the frequency of PPHaV movements between two farms during the 2016–17 HPAI H5N6 epidemic. As a result, 98 IPs of 259 IPs had at least one tie formation with other IPs. First, with those interlinked networks, we examined the relationship between tie formation and the genotype of the HPAI H5N6 virus-infected IPs using a network metric named assortativity. Assortativity is a measure of how likely nodes with the same kind of attribute are to connect each other^21,22^. The assortativity coefficient is positive if similar nodes (i.e., same type of genetic group) tend to connect to each and negative if different genotype infected premises tend to interconnect (see methods in the Supplementary online)^23^. This metric was computed because if high assortativity is present in a network, it suggests homogenous mixing between the same genetic group of IPs, showing high consistency between tie formation via vehicle movement and HPAI transmission chains^22^.

Second, to identify factors contributing to tie formation between the same genotype IPs, we performed logistic regression quadratic assignment permutation (LR-QAP) analysis in which the response variable was the presence (coded as one) or absence (coded as zero) of a tie between two IPs; explanatory variables included poultry integrators, type of farm species, and geographical distance. The type of distance we used was the Euclidean distance between interlinked infected premises via vehicle movement, measured as a continuous scale. We used LR-QAP analysis as a generic methodology for permutation tests in which nodes in the network were randomly re-allocated in the other nodal position of the network, and the test statistic of interest was then calculated and compared with the observed one^24^ because the variable between adjacent nodes was very unlikely to be independent of other network variables and because permutation tests have the advantage of missing data^25^. A* p* value was calculated as the count of permutations that generated statistics more extreme than the observed statistic^25^. As LR-QAP analysis uses dyadic covariates (pairwise variables between two nodes (i.e., IPs)), the explanatory variables were transformed into dyadic values to indicate the difference between the attributes of two nodes. For instance, if node A raised domestic ducks and node B also raised ducks, the value of the corresponding variable for the farm species was converted into zero. Thus, we performed LR-QAP analysis with dyadic covariates and compared the goodness of fit of the final model by comparing the Bayesian information criterion (BIC) values of each network. The R packages 'sna' and ‘statnet' were used to perform LQ-QAP regression^26,27^.

### Bayesian modelling approach

The vehicle movements originating from IPs were hypothesized to be the main sources of infection for domestic poultry holdings. The null model for HPAI H5N6 transmission between the same genotype of IPs would be that the force of infections posed by other sources except for PPHaV movements originating from the same genotype IPs, which is assumed to be zero and results in no contribution of the spread of HPAI. To test this hypothesis, the force of infection was built with two parameters following the approach made in a previous study: $P_{V}$ was the daily risk of infection by one potentially contaminating vehicle movement, and $P_{B}$ was the daily risks of infection other than $P_{V}$. The force of infection for farm *i* on day *d* ($F_{i,d}$) was fomulated as follows:

$$ F_{i,d} = P_{V} n_{i,d}^{V} + P_{B} $$

where $$n_{i,d}^{V}$$ represents the number of potentially contaminating vehicle movements farm *i* received on day *d*. Since dates of HPAI H5N6 infection were not exactly specified for IPs, we updated the infection dates ($$I_{i} )$$, and therefore the time between infection and reporting ($$D_{i} )$$, in each iteration by using a data augmentation approach based on the Metropolis-Hasting (MH) Monte Carlo Markov chain (MCMC) algorithm, which has been applied to infer transmission dynamics from incomplete epidemic data^28–30^. With $$F_{i,d}$$, $$I_{i}$$, and $$D_{i}$$, the likelihood of the epidemic data was expressed as follows:

$$ L = \mathop \prod \limits_{i} {}e^{{{\mathop \sum \nolimits_{d = 1}^{I_{i} - 1}} - F_{i,d} }} \left( {1 - e^{{ - F_{{i,I_{i} }} }} } \right)f\left( {D_{i} ;\alpha ,\beta } \right)\mathop \prod \limits_{j} {}e^{{{\mathop \sum \nolimits_{d = 1}^{d_{{last_{j} }} }} - F_{j,d} }} $$

The first term represents the probability that an IP i remained uninfected until a day before its infection date $I_{i}$ and became infected on day $I_{i}$ and the probability density function of the duration between infection and reporting in farms is expressed as a Gamma distribution, $f\left( {D_{i} ;\alpha ,\beta } \right)$,, with $\alpha$ and $\beta$ as mean and variance. The last term indicates the probability that a non-IP j did not become infected during the study period. The farms were culled during the study period and therefore no longer at risk of infection. Thus, for those farms, $d_{{last_{j} }}$ indicates the date on which the farm was depopulated. We estimated the separate force of infection posed by potentially contaminating vehicle movements originating from four different genotype IPs (i.e., from C2 to C5), assuming that the farms with specific genotypes of the virus only had HPAI H5N6 virus transmitted by the same genotype IPs. Other genotype IPs were regarded as susceptible ones until the reporting date. Consequently, we obtained the joint posterior distribution of model parameters. Given the Gamma distribution by randomly sampling parameters αand βas mean and variance, which was the estimation of the posterior distribution of the interval between infection and reporting, we calculated the contribution of vehicle movements to HPA H5N6 infection in IPs. The more detailed information on the estimation for the contribution of vehicle movements to HPAI infection can be found in the Supplementary method section online.

Three MCMC chains were used with a burn-in of 4,000 iterations and 20,000 iterations were used for each posterior distribution where convergence was inspected by MCMC trace plots and the Gelman-Rubin convergence diagnostic. To assess the best assumption of the infectious duration of potential contaminating vehicle movements (i.e., one day versus three days) explained the epidemic pattern better, we compared models based on their deviance information criterion (DIC) values. Moreover, because infection dates were not observed, we used a modified version of the DIC for models with missing data^31^.

### Permutation analysis on inter-linked same genotype of IPs

To explore contact properties relative to HPAI H5N6 transmission via IPs origin vehicle movements, we built a contact network composed of only the same genotype IPs based on the simulation results. We examined the following six contact properties: diameter, average path length, density, reciprocity, assortativity, and transitivity. The diameter of a network is defined as the shortest distance between the two most distant nodes in the network. The average path length is equal to the number of edges along the its shortest length between two nodes. Density is the proportion of edges that exist in a network. Reciprocity is the proportion of nodes in a directed network to be mutually linked. Transitivity refers to the ratio between the observed closed triplets and the maximum possible number of closed triplets in the graph. This index indicates how many tightly interconnected communities existed in a network^32^. The farm species and degree that represents the number of connections were used to measure the assortativity of the network as aforementioned. We calculated these metrics for directed and weighted networks using the R package "i-graph"^33^. Subsequently, we tested hypotheses about whether these metrics of observed contact networks between IPs were statistically different from those of simulated contact structure, which is a possible indicator of the epidemiological relevance of HPAI H5N6 transmission events^34^. Hypothesis testing was performed with a permutation approach where 1000 random networks were generated based on the density and the number of nodes. The test statistic, which included six contact properties, was calculated on the random network and compared with those of the observed network^25,35,36^. The median difference between observed value and simulated values with 5–95% percentile was calculated as the right skewed distribution. The C5-genotype IP interlinked network was excluded from the permutation test because of the low number of vertices and edges in a cluster. All maps in the figures present manuscript and supplementary file were generated based on the map obtained from the National Spatial Information Portal in the Republic of Korea that was publicly available (http://data.nsdi.go.kr/dataset/12942) using R software version 4.0.4.

## Supplementary Information

Supplementary Information.

## Data Availability

The datasets analysed during the current study are publicly available in the https://home.kahis.go.kr/home/lkntscrinfo/selectLkntsOccrrncList.do except for vehicle movement data (GPS) due to privacy policy, ROK.

## References

[1] Dhingra MS (2018). Geographical and historical patterns in the emergences of novel highly pathogenic avian influenza (HPAI) H5 and H7 viruses in poultry. Front. Vet. Sci..

[2] Hagenaars TJ, Boender GJ, Bergevoet RHM, van Roermund HJW (2018). Risk of poultry compartments for transmission of Highly Pathogenic Avian Influenza. PLoS ONE.

[3] Lee H-J (2014). Prediction of the spread of highly pathogenic avian influenza using a multifactor network: Part 2 – Comprehensive network analysis with direct/indirect infection route. Biosys. Eng..

[4] Sun X (2018). Social network analysis for poultry HPAI transmission. Transbound. Emerg. Dis..

[5] Guinat C (2020). Role of live-duck movement networks in transmission of avian influenza, France, 2016–2017. Emerg. Infect. Dis..

[6] Jeong, M., Jang, I.-H. & Choe, Y. Network Analysis of Swine Farms and Slaughters: Based on Automobile GPS Data. (2019).

[7] Lee EK (2017). Multiple novel H5N6 highly pathogenic avian influenza viruses, South Korea, 2016. Infect. Genet. Evol..

[8] Bataille A, van der Meer F, Stegeman A, Koch G (2011). Evolutionary analysis of inter-farm transmission dynamics in a highly pathogenic avian influenza epidemic. PLoS Pathog..

[9] Seo, J. in *Korean poultry journal* Vol. November 120–122 (Korean poultry association, 2013).

[10] Agency, A. a. P. Q. (ed Veterinary epidemiology) 31 (Ministry of Agriculture, Food and Rural Affairs 2018).

[11] Fasina FO, Rivas AL, Bisschop SP, Stegeman AJ, Hernandez JA (2011). Identification of risk factors associated with highly pathogenic avian influenza H5N1 virus infection in poultry farms, in Nigeria during the epidemic of 2006–2007. Prev. Vet. Med..

[12] Thomas ME (2005). Risk factors for the introduction of high pathogenicity Avian Influenza virus into poultry farms during the epidemic in the Netherlands in 2003. Prev. Vet. Med..

[13] 821–843 (World Organisation for Animal Health Organization, World Organisation for Animal Health, 2018).

[14] Takemae N (2017). Five distinct reassortants of H5N6 highly pathogenic avian influenza A viruses affected Japan during the winter of 2016–2017. Virology.

[15] Si YJ (2017). Genetic characterisation of novel, highly pathogenic avian influenza (HPAI) H5N6 viruses isolated in birds, South Korea, November 2016. Euro Surveill..

[16] Guinat C (2014). Dynamics of African swine fever virus shedding and excretion in domestic pigs infected by intramuscular inoculation and contact transmission. Vet. Res..

[17] Agency, A. a. P. Q. 42–43 (Ministry of Agriculture, Food and Rural Affairs 2018).

[18] Spackman E, Cattoli G, Suarez DL (2016). Diagnostics and surveillance methods. Animal Influenza.

[19] Wood JP, Choi YW, Chappie DJ, Rogers JV, Kaye JZ (2010). Environmental persistence of a highly pathogenic avian influenza (H5N1) virus. Environ. Sci. Technol..

[20] Swayne DE (2016). Animal influenza.

[21] Newman ME (2003). Mixing patterns in networks. Phys. Rev. E.

[22] Moyen N (2018). A large-scale study of a poultry trading network in Bangladesh: implications for control and surveillance of avian influenza viruses. BMC Vet. Res..

[23] Hao D, Li C (2011). The dichotomy in degree correlation of biological networks. PLoS ONE.

[24] Borgatti SP, Everett MG, Johnson JC (2018). Analyzing social networks.

[25] VanderWaal K, Enns EA, Picasso C, Packer C, Craft ME (2016). Evaluating empirical contact networks as potential transmission pathways for infectious diseases. J. R. Soc. Interface.

[26] Butts C. T. sna: Tools for Social Network Analysis. R package version 2.4, https://CRAN.R-project.org/package=sna (2016).

[27] Handcock MS, Hunter DR, Butts CT, Goodreau SM, Morris M (2008). statnet: Software tools for the representation, visualization, analysis and simulation of network data. J. Stat. Softw..

[28] Cauchemez S (2011). Role of social networks in shaping disease transmission during a community outbreak of 2009 H1N1 pandemic influenza. Proc. Natl. Acad. Sci..

[29] Walker PGT (2010). A Bayesian approach to quantifying the effects of mass poultry vaccination upon the spatial and temporal dynamics of H5N1 in Northern Vietnam. PLoS Comput. Biol..

[30] Chapman LAC (2018). The role of case proximity in transmission of visceral leishmaniasis in a highly endemic village in Bangladesh. PLoS Negl. Trop. Dis..

[31] Celeux G, Forbes F, Robert CP, Titterington DM (2006). Deviance information criteria for missing data models. Bayesian Anal..

[32] Lusher D, Koskinen J, Robins G (2013). Exponential random graph models for social networks: theory, methods, and applications.

[33] Csardi G, Nepusz T (2006). The igraph software package for complex network research. InterJournal Complex Syst..

[34] Van Borkulo, C. D. *et al.* Comparing network structures on three aspects: A permutation test. *Manuscript submitted for publication*. 10–11 (2017).

[35] Kiss IZ, Green DM, Kao RR (2006). The network of sheep movements within Great Britain: Network properties and their implications for infectious disease spread. J. R. Soc. Interface.

[36] Farine DR (2017). A guide to null models for animal social network analysis. Methods Ecol. Evol..

