# Supplementary material for: Dynamics of inter-farm transmission of highly pathogenic avian influenza H5N6 integrating vehicle movements and phylogenetic information
Source: Sci Rep. 2021 Dec 17;11:24163. doi: 10.1038/s41598-021-03284-x (PMC8683487; doi:10.1038/s41598-021-03284-x)
Supplement: Supplementary file 1 — Supplementary Information. [file 41598_2021_3284_MOESM1_ESM.docx]

Supplementary Information

**Methods**

*Network analysis*

An assortativity is a measure of how much the nodes that possess the same kind of attribute are more likely to connect each other, which is written as follows^1,2^

$$assortativity = (\sum_{i} e_{ii}-\sum_{i} a_{i}b_{i})/ (1-\sum_{i} a_{i}b_{i})$$

$$a_{i} = \sum_{j} e_{ij}, b_{j}=\sum_{i} e_{ij}$$

Where i indicate type, e_ii_ is the fraction of edges connecting a node of type i and a node of type i (i.e., same type edge, e.g., IP of genotype i) is summed to one. a_i_ and b_i_ are the portion of each type (e.g., genotype) of end of an edge that is attached to nodes of type i. If the same type to type connection is solely present in the network, sum e_ii_ =1, sum a_i_=0 and sum b_i_=0 result in assortativity of one. The assortativity coefficient is positive if similar node (based on some external property) tends to connect to each, and negative otherwise ^3^.

*Bayesian inference*

We estimated the posterior distribution of the length of time between infection and reporting through simulations based on the joint posterior distribution of $\alpha$ and $\beta$.Given the Gamma distribution by randomly sampling parameters $\alpha$ and $\beta$ as mean and variance which was estimation of the posterior distribution of the interval between infection and reporting, we calculated the probability that IP *i* was infected on day *d*, $\delta\left( i, d \right)$, and the probability that IP *j* was infectious on day *s*, $\pi\left( j, s \right)$. If vehicle *v* exited from IP *j* on day *s* and entered IP *i* on day *d*, the force of infection by vehicle movement ($F_{V,j,s,i,d}$) was evaluated as follows:

$$F_{V,j,s,i,d}=\delta\left( i, d \right)\pi\left( j,s \right)P_{V}I(s,d)$$

Where $I(s,d)$ was equal to 1 if the time difference between days *s* and *d* was less than the pre-defined infectious duration of PPHaV, and 0 otherwise. The force of infection $F_{V,i}$ posed by vehicle movements originating from infectious IP *i* was estimated as follows:

$$F_{V,i}=\sum_{V,j,s,d} F_{V,j,s,i,d}$$

In each iteration, the type of source of infection was assigned to each IP *i* by simulating a binomial trial, with one trial and two possible outcomes (i.e., vehicle movement and other source) composed of probabilities $F_{V,i}/K$, and $F_{B,i}/K$, with $K=F_{V,i}+F_{B,i}$. $F_{B,i}$ , the force of infection posed by the other sources was equal to $P_{B}$. This process was repeated for 10,000 times and the proportion of simulated outcome of infection posed by each transmission route was measured

| **Table S1.** Priors for the force of infection and Gamma distribution parameters | | | |
| --- | --- | --- | --- |
| Parameters | Priors | | |
| Force of infection parameters | | P_v_ | Uniform (0, 1) |
|  |  | P_B_ | Uniform (0, 1) |
| Gamma distribution parameters^*^ | | α | Uniform (1, 20) |
|  |  | β | Gamma (mean = 6.5, variance = 2) |
| * α and β were the mean and variance of the Gamma distribution of the time between infection and reporting. | | | |

**Table S2.** List of poultry production and health associated vehicles (PPHaVs) (n=18)

| Type of PPHaV | Description |
| --- | --- |
| Feed transporter | A vehicle to supply feeding materials other than roughage to livestock holdings |
| Animal transporter | A vehicle to transport animals to livestock holdings |
| Manure Hauling | A vehicle to collect manure of livestock holdings |
| Egg transporter | A vehicle to collect eggs of poultry holdings |
| Private veterinarian | A vehicle for veterinary treatment |
| Veterinary medicine vendor | A vehicle to supply veterinary medicine to livestock holdings |
| Consultant | A vehicle used by livestock farming consultant |
| Bedding material supplier | A vehicle to supply bedding materials including wood shavings, saw dust, rice hull and straw to livestock holdings |
| Compost Transporter | A vehicle to collect composted produced by livestock holdings |
| Repair service | A vehicle to repair materials and facilities in livestock holdings |
| Milk transporter | A vehicle to collect milk produced dairy cows in livestock holdings |
| Artificial insemination service | A vehicle to collect milk produced dairy cows in livestock holdings |
| Egg flats transporter | A vehicle to supply egg rack and flats to poultry holdings |
| Live haul loaders | A vehicle for load-out crew, catch crew |
| Veterinary service | A vehicle for veterinary services by animal health authorities |
| Poultry by-product transporter | A vehicle to collect and transport poultry by-product |
| Livestock-farming facility management | A vehicle used for other purpose by farmworkers in livestock holdings |
| Roughage feed truck | A vehicle to supply roughage to livestock holdings |

| **Results**  **Table S3.** Posterior parameter estimates and posterior predictive length of time between infection and reporting from infected premises of each phylogenetic cluster, with different assumptions on the duration of vehicle infectiousness | | | | | |
| --- | --- | --- | --- | --- | --- |
| Parameter | HPAI H5N6 phylogenetic cluster | | | | |
|  | C2 (no. cases=81) | C3 (no. cases=41) | C4 (no. cases=114) | C5 (no. cases=23) | |
| Duration of vehicle infectious: One day |  |  |  |  | |
| No. of vehicle movements | 528 (100%) | 523 (100%) | 838 (100%) | 262 (100%) | |
| Between IPs | 53 (10.0%) | 130 (24.9%) | 207 (24.7%) | 3 (1.1%) | |
| IPs to non-IPs | 475 (90.0%) | 393 (75.1%) | 631 (75.35) | 259 (98.9%) | |
| Potentially contaminating vehicle movement* | 37.25 x10^-4^ (0.01, 159.08) x10^-4^ | 60.18 x10^-4^ (0.04, 260.28) x10^-4^ | 34.58 x10^-4^ (0.01, 147.46) x10^-4^ | 105.56 x10^-4^  (0.04, 463.04) x10^-4^ | |
| Other sources* | 0.01 x10^-4^ (0, 0.04) x10^-4^ | 0.01 x10^-4^ (0, 0.05) x10^-4^ | 0.01 x10^-4^ (0, 0.04) x10^-4^ | 0.01 x10^-4^ (0, 0.04) x10^-4^ | |
| Mean of the Gamma distribution* | 8.30  (1.72, 13.84) | 7.46  (1.20,13.49) | 7.52  (1.16, 13.49) | 7.52  (1.46, 8.34) | |
| Variance of the Gamma distribution* | 42.66  (9.84, 117.77) | 41.89  (5.36,109.24) | 41.62  (9.06, 114.23) | 41.42  (8.34, 114.33) | |
| DIC† | 1519.98 | 1347.80 | 4014.53 | 808.85 | |
| Duration of vehicle infectious: Three days |  |  |  |  | |
| No. of vehicle movements | 2,173 (100%) | 2,038 (100%) | 2,958 (100%) | 913 (100%) | |
| Between IPs | 230 (10.6%) | 566 (27.8%) | 529 (17.9%) | 13 (1.4%) | |
| IPs to non-IPs | 1,943 (89.4%) | 1,472 (72.2%) | 2,429 (82.1%) | 900 (98.6%) | |
| Potentially contaminating vehicle movement* | 16.58 x10^-4^ (0, 69.88) x10^-4^ | 21.15 x10^-4^  (0, 87.93) x10^-4^ | 11.38 x10^-4^ (0, 46.70) x10^-4^ | 41.02 x10^-4^ (0.01, 190.83) x10^-4^ | |
| Other sources* | 0.01 x10^-4^ (0, 0.04) x10^-4^ | 0.01  (0, 0.04) x10^-4^ | 0.01 (0, 0.04) x10^-4^ | 0.01 (0, 0.04) x 10^-4^ | |
| Mean of the Gamma distribution* | 7.45  (1.26, 13.55) | 7.64  (1.65, 13.99) | 7.45  (1.69, 13.99) | 7.64 (1.43, 13.68) | |
| Variance of the Gamma distribution* | 41.62  (9.03, 116.99) | 41.42  (8.50, 113.09) | 42.24  (9.77, 119.89) | 41.53  (9.02, 114.18) | |
| DIC† | 2812.28 | 1386.40 | 3938.62 | 808.69 | |
| * The value expressed by median with 95% highest density interval (parenthesis) of posterior marginal distribution,  † The deviance information criteria. DIC | | | | |  |


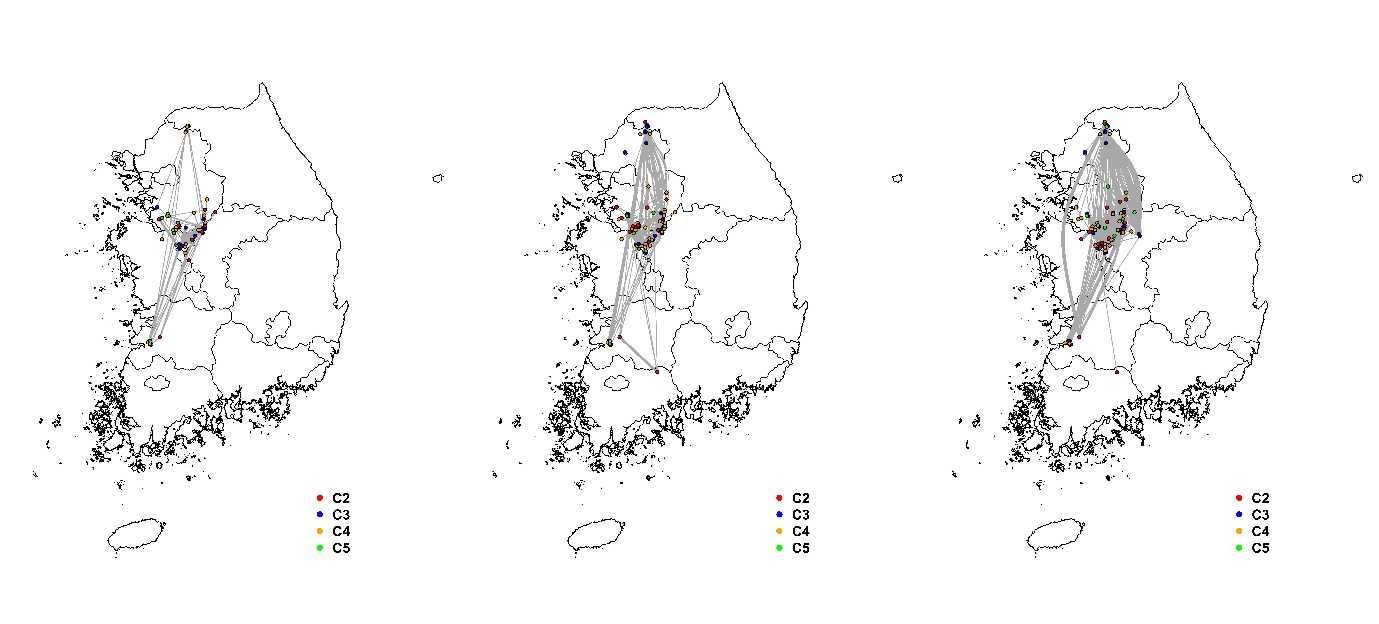


**Figure S1. Spatial representation of contact network consisting of all genotypes of IPs, constructed by under different temporal assumption on infectious duration for IPs and one day duration for vehicle movements.** From left to right, 7days, 14 days and 21 days infectious periods for IPs.


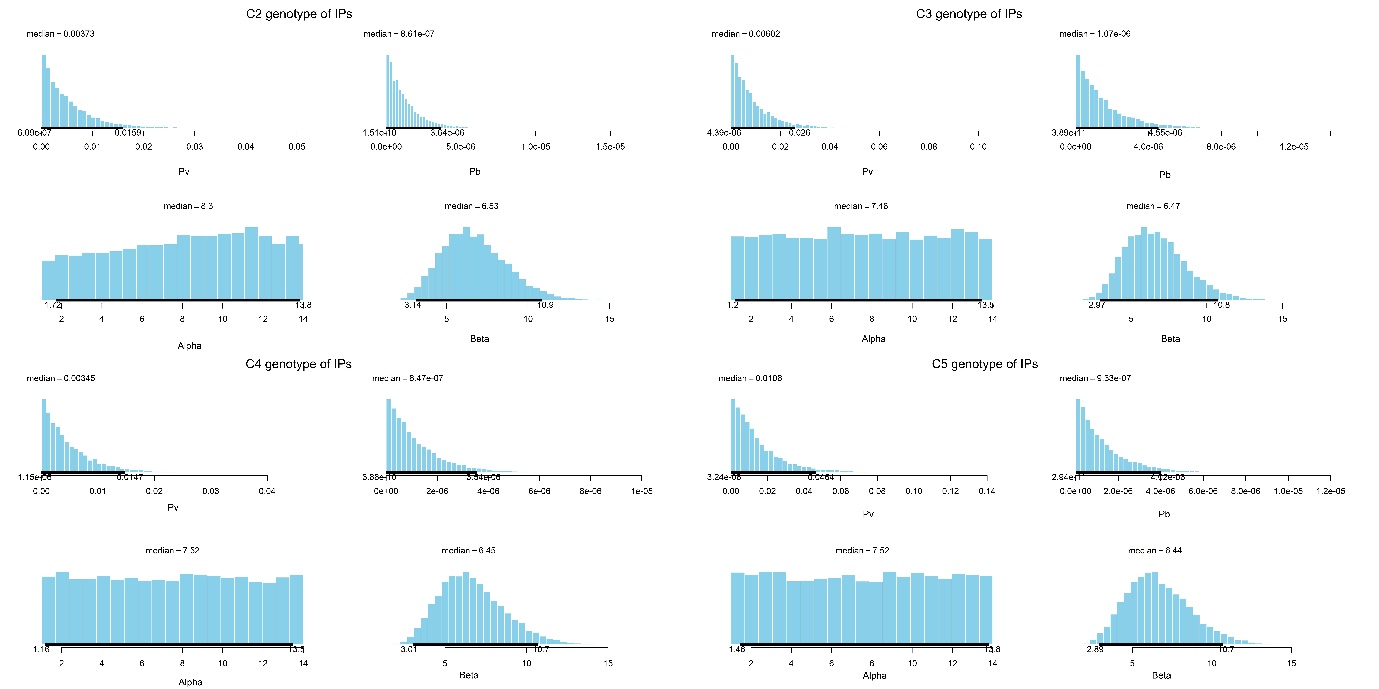


**Figure S2.** Posterior distribution of parameters relating to potentially contaminating vehicle movement and other sources of infections, and interval length between infection and reporting of HPAI based on the infection duration of vehicle movement as one day.


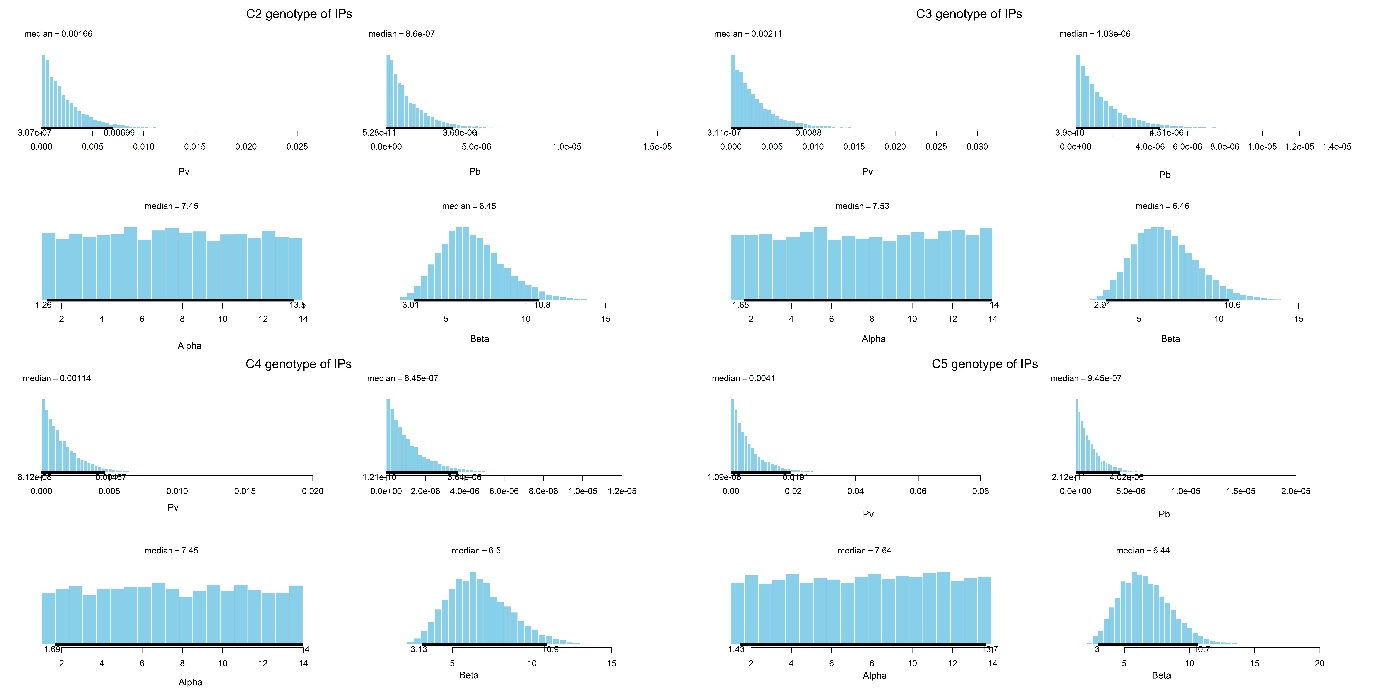


**Figure S3.** Posterior distribution of parameters relating to potentially contaminating vehicle movement and other sources of infections, and interval length between infection and reporting of HPAI based on the infection duration of vehicle movement as three days.


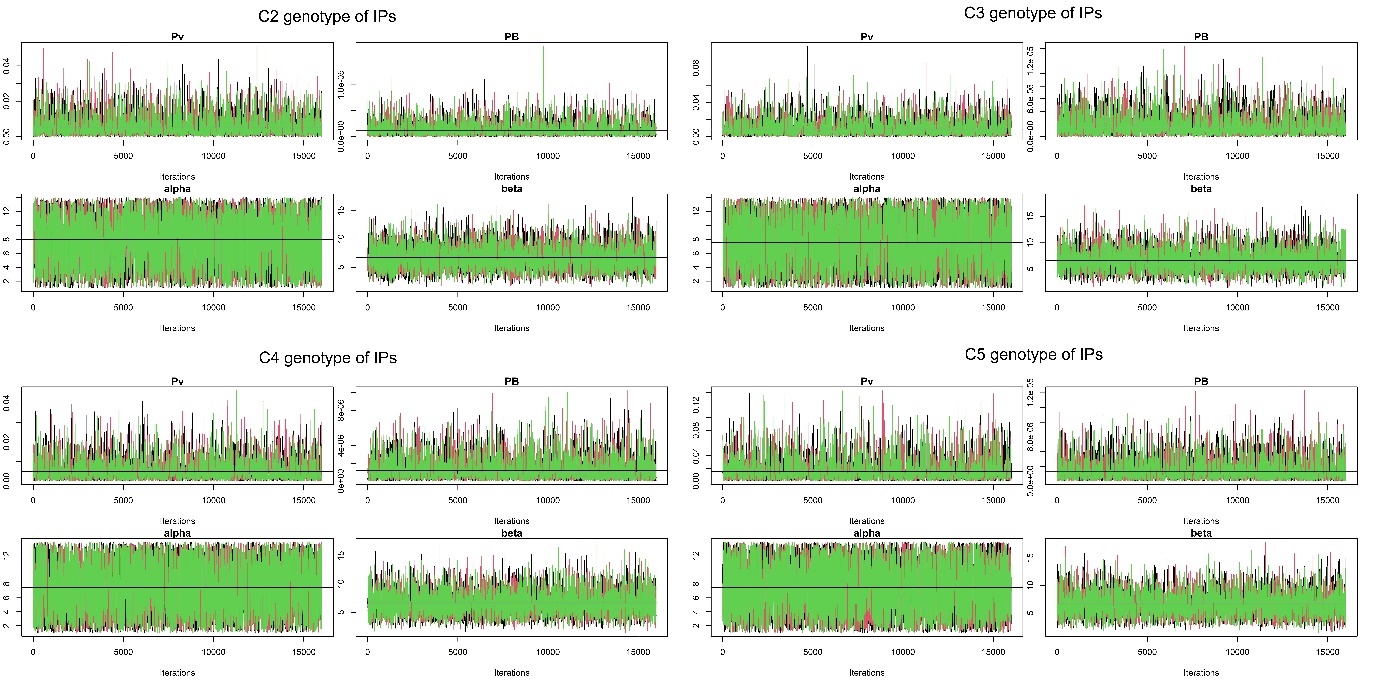


**Figure S4** Trace plot for posterior marginal distribution of the parameters relating to potentially contaminating vehicle movement and other sources of infections, and interval length between infection and reporting of HPAI based on the infection duration of vehicle movement as one day. All parameters converged, with Gelman-Rubin statistics less than 1.01.

**
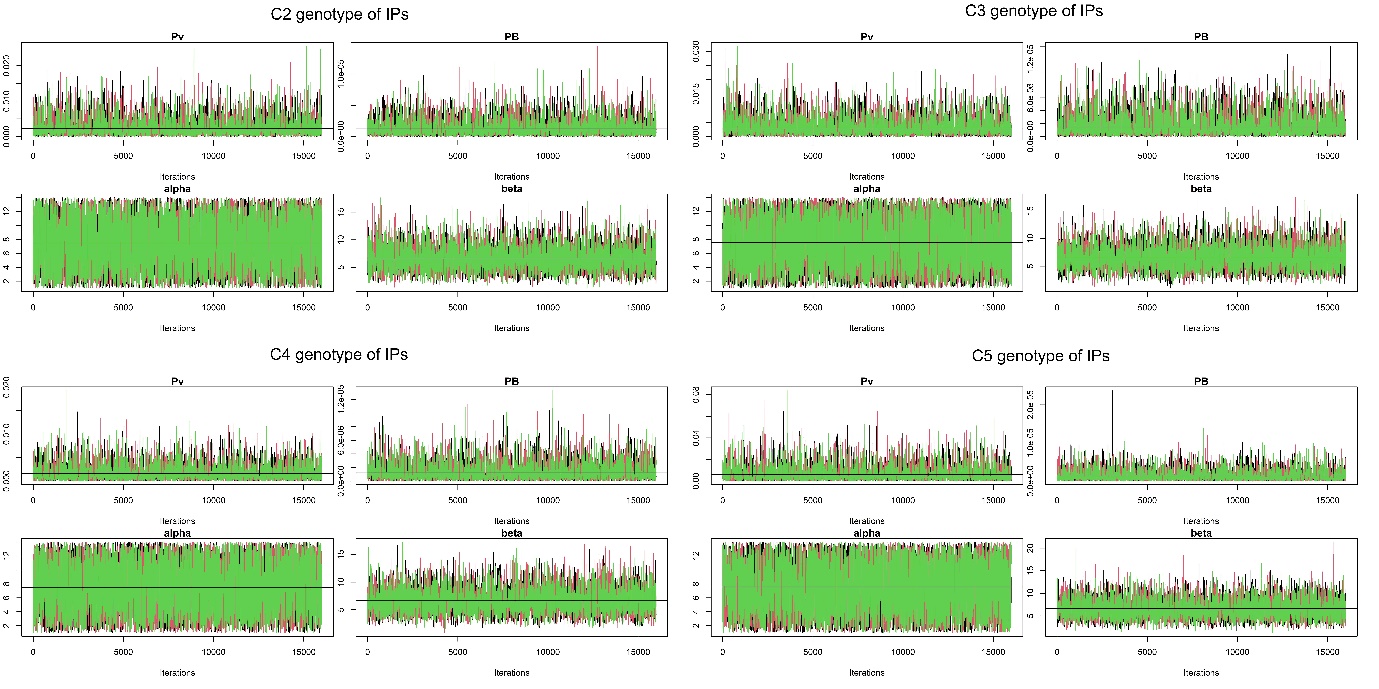
**

**Figure S5** Trace plot for posterior marginal distribution of the parameters relating to potentially contaminating vehicle movement and other sources of infections, and interval length between infection and reporting of HPAI based on the infection duration of vehicle movement as three days. All parameters converged, with Gelman-Rubin statistics less than 1.01.

**
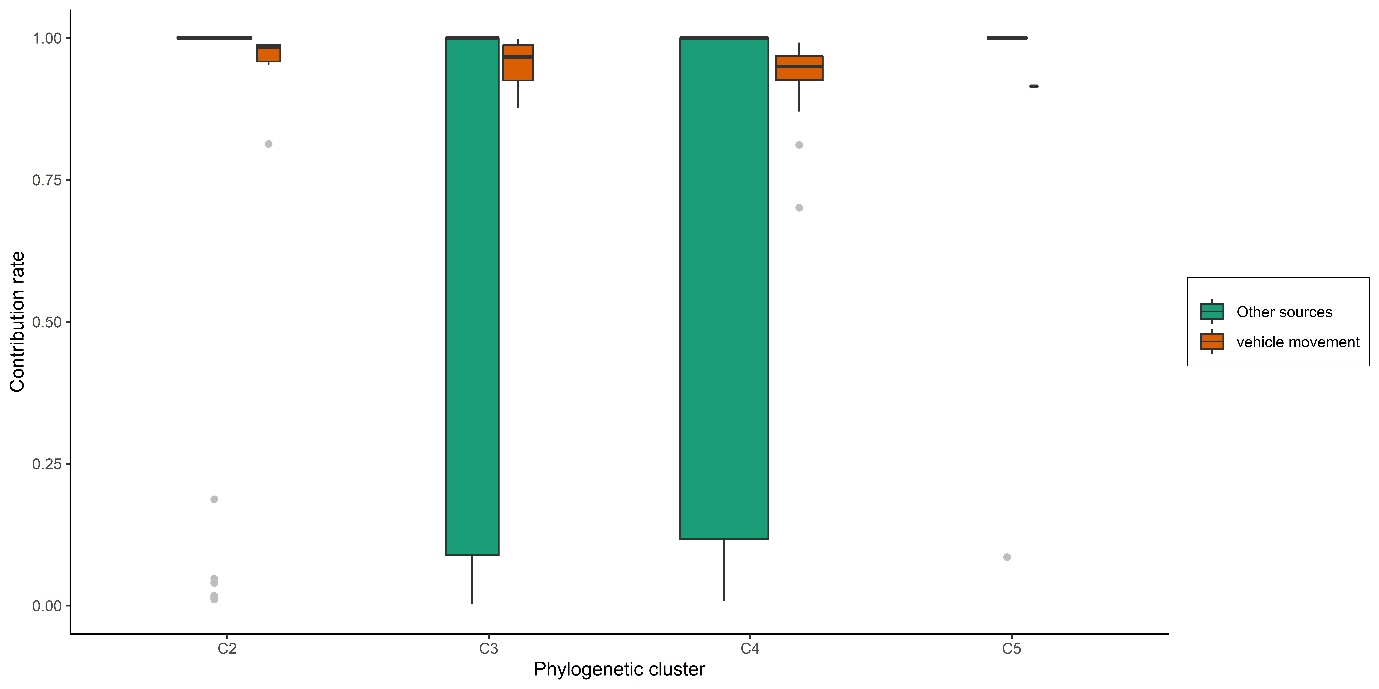
**

**Figure S6** Boxplot for distribution of contribution rate of both potentially contaminating vehicle movement that lasted the infectious duration of one day and other sources to HPAI H5N6 infection in infected premises.

**
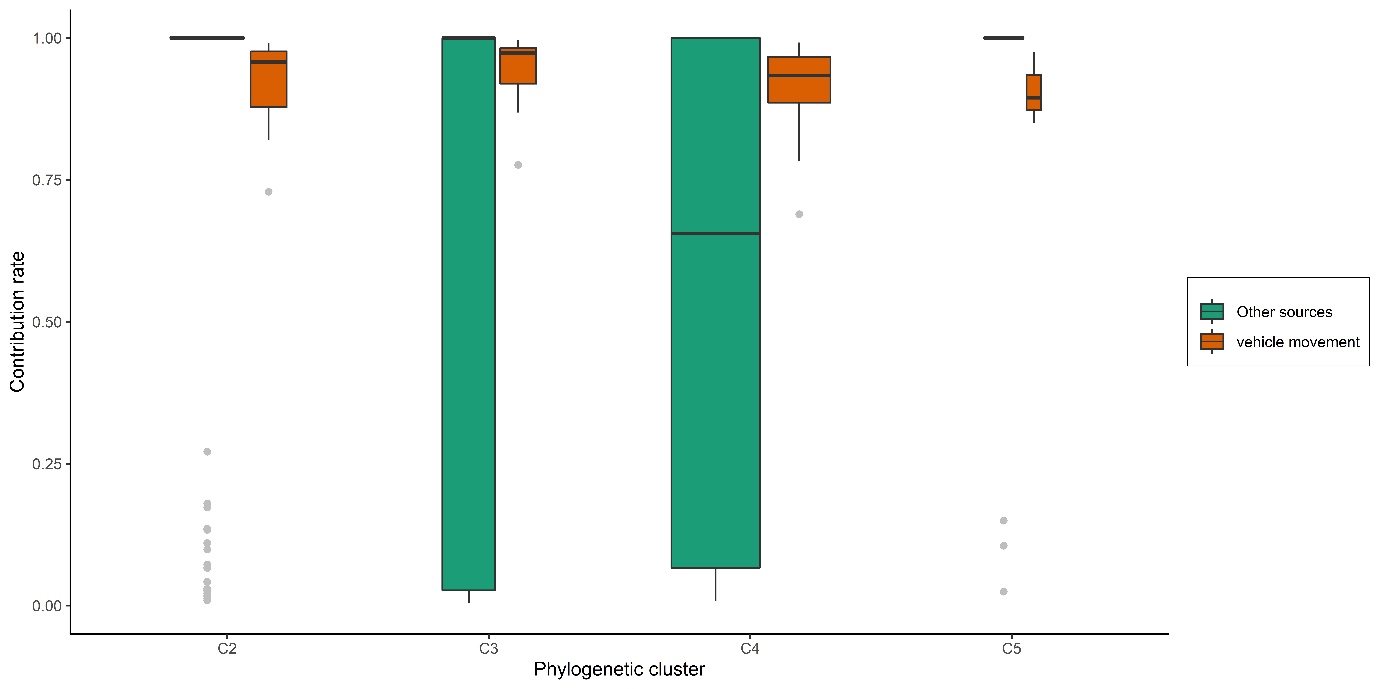
**

**Figure S7** Boxplot for distribution of contribution rate of both potentially contaminating vehicle movement that lasted the infectious duration of three days and other sources to HPAI H5N6 infection in infected premises.


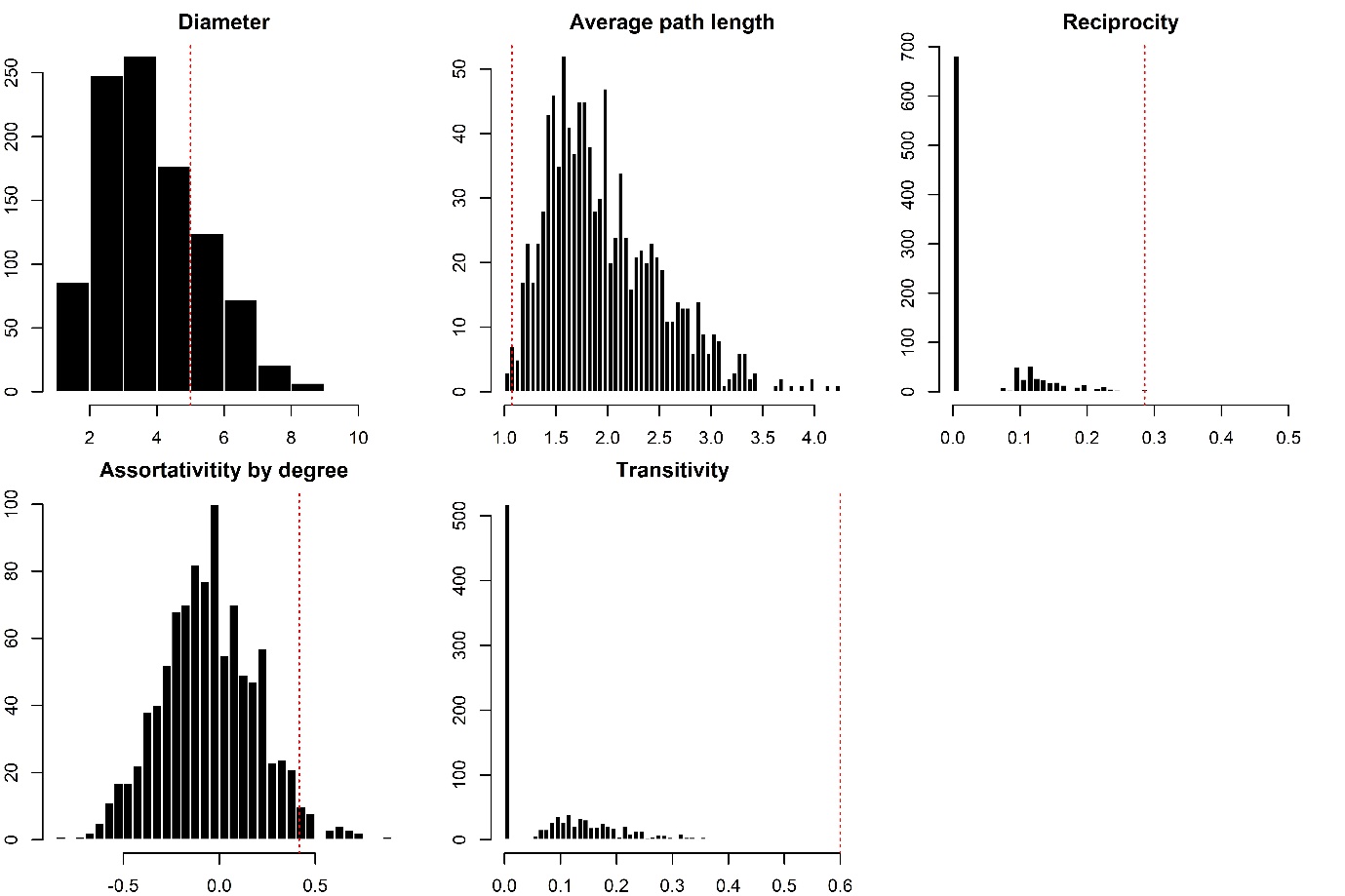


**Figure S8. Distribution of permutated test statistics versus and observed value in C2 contact network.** A red dotted line denotes the observed values.

**
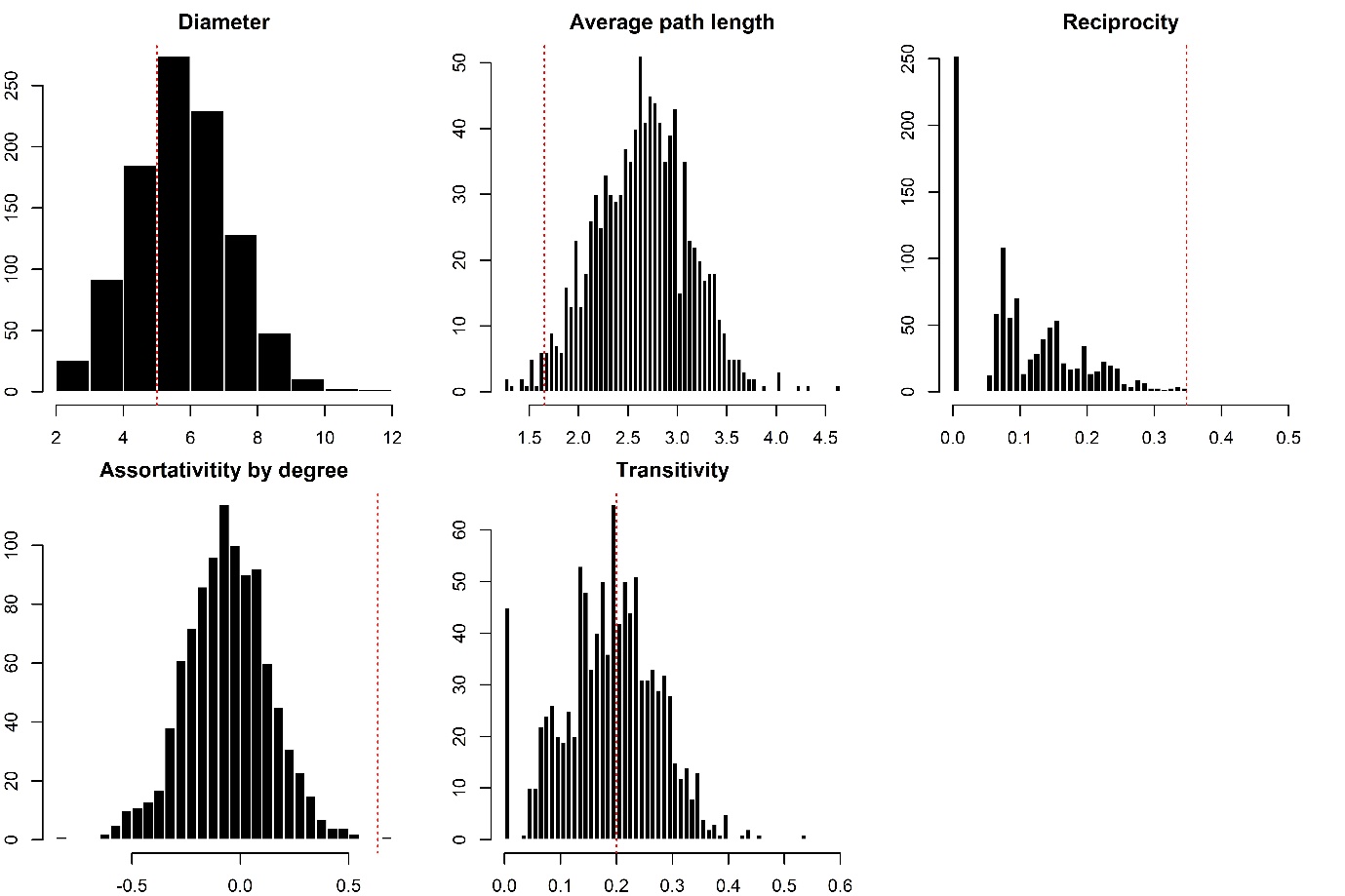
**

**Figure S9. Distribution of permutated test statistics versus and observed value in C3 contact network.** A red dotted line denotes the observed value.


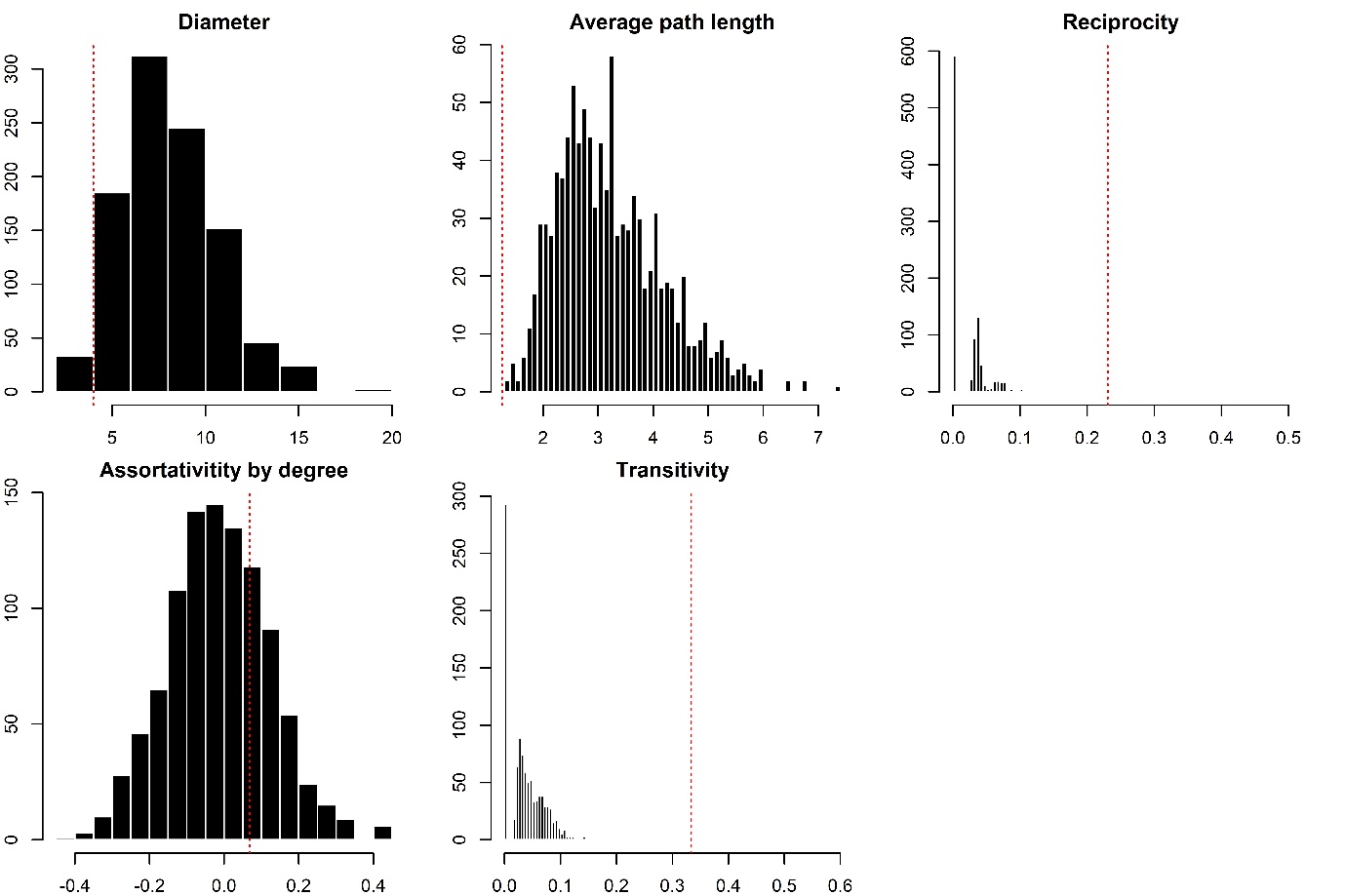


**Figure S10. Distribution of permutated test statistics versus and observed value in C4 contact network.** A red dotted line denotes the observed value.


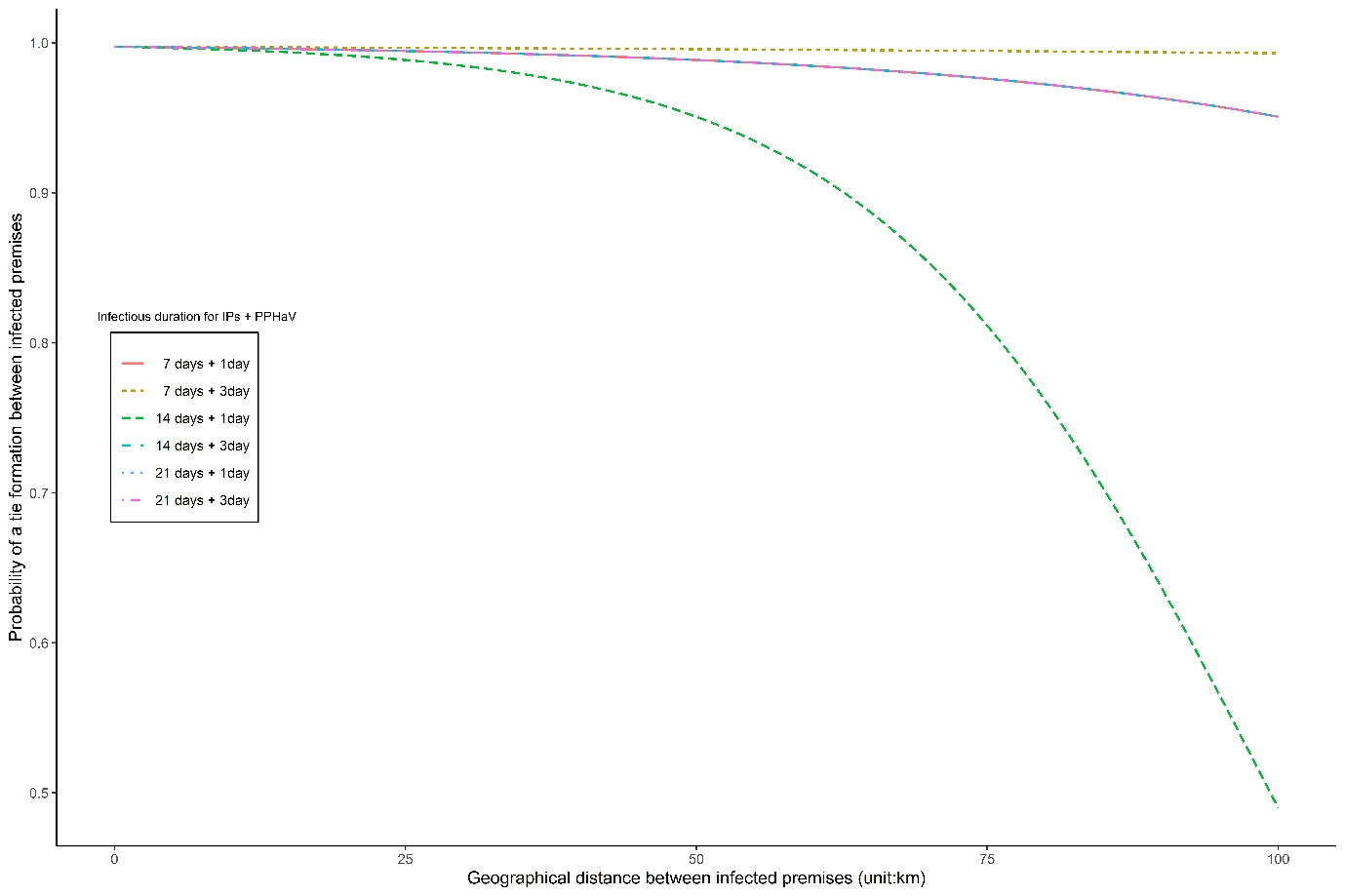


**Figure** **S11** The estimated probability of a tie formation between HPAI H5N6 virus-infected premises via vehicle movements over the geographical distance between infected premises given


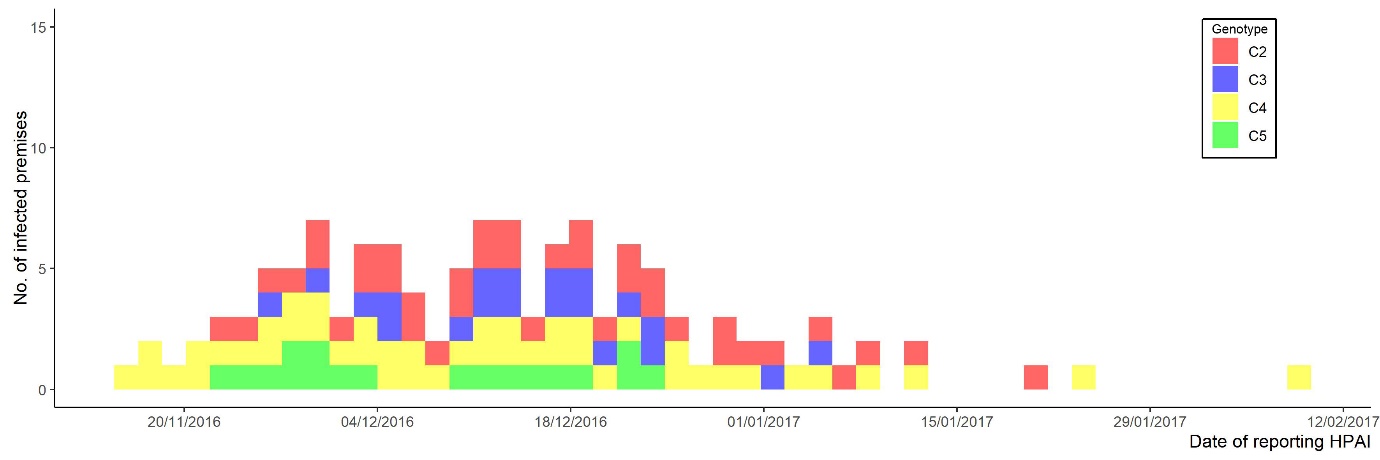


**Figure S12** Temporal distribution of reporting date of four HPAI H5N6 phylogenetic clusters of 259 infected premises used in Bayesian inference

**References**

1. Newman, M.E. Mixing patterns in networks. *Physical review E* **67**, 026126 (2003).

2. Moyen, N.*, et al.* A large-scale study of a poultry trading network in Bangladesh: implications for control and surveillance of avian influenza viruses. *BMC veterinary research* **14**, 12 (2018).

3. Hao, D. & Li, C. The dichotomy in degree correlation of biological networks. *PLoS One* **6**, e28322 (2011).
